# Unleashing T cell anti-tumor immunity: new potential for 5-Nonloxytryptamine as an agent mediating MHC-I upregulation in tumors

**DOI:** 10.1186/s12943-023-01833-8

**Published:** 2023-08-15

**Authors:** Paweł Stachura, Wei Liu, Haifeng C. Xu, Agnès Wlodarczyk, Olivia Stencel, Piyush Pandey, Melina Vogt, Sanil Bhatia, Daniel Picard, Marc Remke, Karl S. Lang, Dieter Häussinger, Bernhard Homey, Philipp A. Lang, Arndt Borkhardt, Aleksandra A. Pandyra

**Affiliations:** 1 Department of Molecular Medicine II, Medical Faculty, Heinrich-Heine-University, Universitätsstraße 1, 40225 Düsseldorf, Germany; 2 Department of Pediatric Oncology, Hematology and Clinical Immunology, Medical Faculty, Center of Child and Adolescent Health, Heinrich-Heine-University, Moorenstrasse 5, 40225 Düsseldorf, Germany; 3 Division of Pediatric Neuro-Oncogenomics, German Cancer Research Center (DKFZ), Heidelberg, Germany; 4 Partner Site Essen/Düsseldorf, German Consortium for Translational Cancer Research (DKTK), Düsseldorf, Germany; 5 Department of Neuropathology, Medical Faculty, Heinrich-Heine University, Moorenstrasse 5, Düsseldorf, 40225 Germany; 6 Institute of Immunology, Medical Faculty, University of Duisburg-Essen, Hufelandstrasse 55, 45147 Essen, Germany; 7 Department of Gastroenterology, Hepatology and Infectious Diseases, Medical Faculty, Heinrich-Heine-University, Moorenstrasse 5, Düsseldorf, 40225 Germany; 8 Department of Dermatology, Medical Faculty, Heinrich-Heine-University, Moorenstrasse 5, Düsseldorf, 40225 Germany; 9 Institute of Clinical Chemistry and Clinical Pharmacology, University Hospital Bonn, Venusberg-Campus 1, 53127 Bonn, Germany; 10 German Center for Infection Research (DZIF), Partner Site Bonn-Cologne, Bonn, Germany

**Keywords:** CD8^+^ T cells, Immunotherapy, Antigen-presenting machinery, 5-Nonyloxytryptamine (5-NL), Cold tumors, cAMP response element-binding protein (CREB)

## Abstract

**Background:**

New therapies are urgently needed in melanoma, particularly in late-stage patients not responsive to immunotherapies and kinase inhibitors. To uncover novel potentiators of T cell anti-tumor immunity, we carried out an ex vivo pharmacological screen and identified 5-Nonyloxytryptamine (5-NL), a serotonin agonist, as increasing the ability of T cells to target tumor cells.

**Methods:**

The pharmacological screen utilized lymphocytic choriomeningitis virus (LCMV)-primed splenic T cells and melanoma B16.F10 cells expressing the LCMV gp33 CTL epitope. In vivo tumor growth in C57BL/6 J and NSG mice, in vivo antibody depletion, flow cytometry, immunoblot, CRISPR/Cas9 knockout, histological and RNA-Seq analyses were used to decipher 5-NL’s immunomodulatory effects in vitro and in vivo.

**Results:**

5-NL delayed tumor growth in vivo and the phenotype was dependent on the hosts’ immune system, specifically CD8^+^ T cells. 5-NL’s pro-immune effects were not directly consequential to T cells. Rather, 5-NL upregulated antigen presenting machinery in melanoma and other tumor cells in vitro and in vivo without increasing PD-L1 expression. Mechanistic studies indicated that 5-NL’s induced MHC-I expression was inhibited by pharmacologically preventing cAMP Response Element-Binding Protein (CREB) phosphorylation. Importantly, 5-NL combined with anti-PD1 therapy showed significant improvement when compared to single anti-PD-1 treatment.

**Conclusions:**

This study demonstrates novel therapeutic opportunities for augmenting immune responses in poorly immunogenic tumors.

**Supplementary Information:**

The online version contains supplementary material available at 10.1186/s12943-023-01833-8.

## Introduction

The incidence of melanoma and mortality is on the rise and despite therapeutic advances, the 3-year survival low [1, 2]. Since the advent of immunotherapies such as ipilimumab [3], nivolumab and pembrolizumab [4, 5], the overall survival in patients with advanced melanoma has improved. Combining immunotherapeutic agents with each other when functionally non-redundant or tyrosine kinase targeting agents is a promising approach to overcome resistance associated with the application of single therapies [6]. However, due to toxicities as well as high costs [7, 8], it remains to be determined whether it’s a feasible long-term strategy especially for patients receiving treatment in poorly funded health care settings. Taken together, there is a need to explore novel and more cost-effective treatment options that can enhance the activity of current immunotherapies.

Well characterized mediators of immune-directed tumor cell killing are cytotoxic T lymphocytes (CTLs). CTLs activation occurs through interaction of the T cell receptor-cluster of differentiation 3 (TCR-CD3) complex [9] present on the surface of T cells with peptides loaded onto the major histocompatibility complex class I (MHC-I) on antigen presenting cells (APCs) [10]. Effector CTLs can exert antigen-driven anti-tumor responses through granule exocytosis mediated by perforin and the granule-associated enzymes (granzymes), through Fas ligand (FasL) induced apoptosis, or indirectly through secreted cytokines such as interferon γ (IFNγ) [11, 12].

Therapeutic efforts to boost anti-tumor CD8^+^ T cell immunity have focused on manipulating several aspects of CTL function including re-activation of exhausted CTLs (anti-PD1, LAG3 and TIM3 monoclonal antibodies) [6], expansion of highly reactive tumor infiltrating T cells (Adoptive Cell Transfer Immunotherapy) [13], boosting tumor antigen specific T cell responses (cancer vaccines employing neoantigens and tumor associated antigens) [14] and boosting CTL priming (CD27 agonists) [15]. Taken together, improving CTL function is a promising therapeutic approach with already apparent significant clinical benefits to late stage melanoma patients. Some major obstacles driving immune evasion and hampering immunotherapy responses include a poorly infiltrated ‘cold’ tumor microenvironment (TME), a heterogenous immunosuppressive TME, low mutational burden and silencing of MHC-I or other parts of the antigen-presenting machinery [16].

The lymphocytic choriomeningitis virus (LCMV) is a prototypic arenavirus that has been used for decades to study CD8^+^ effector T cell responses. LCMV’s experimental use has led to important discoveries such as programmed cell death protein 1 (PD1) and its role in T cell exhaustion [17]. Expression of LCMV-specific epitopes on tumor cells facilitates the study of various aspects of CD8^+^ T cell mediated anti-tumor immunity [18, 19]. In our current study,we used B16.F10 cells expressing the H-2Db-restricted GP33 peptide (B16.GP33) CTL epitope [18] to uncover novel agents capable of augmenting T cell responses against tumor cells.

## Results

### Pharmacological screening identifies 5-Nonyloxytryptamine (5-NL) as potentiating anti-tumor immunity in vitro and in vivo

**Fig. 1:**
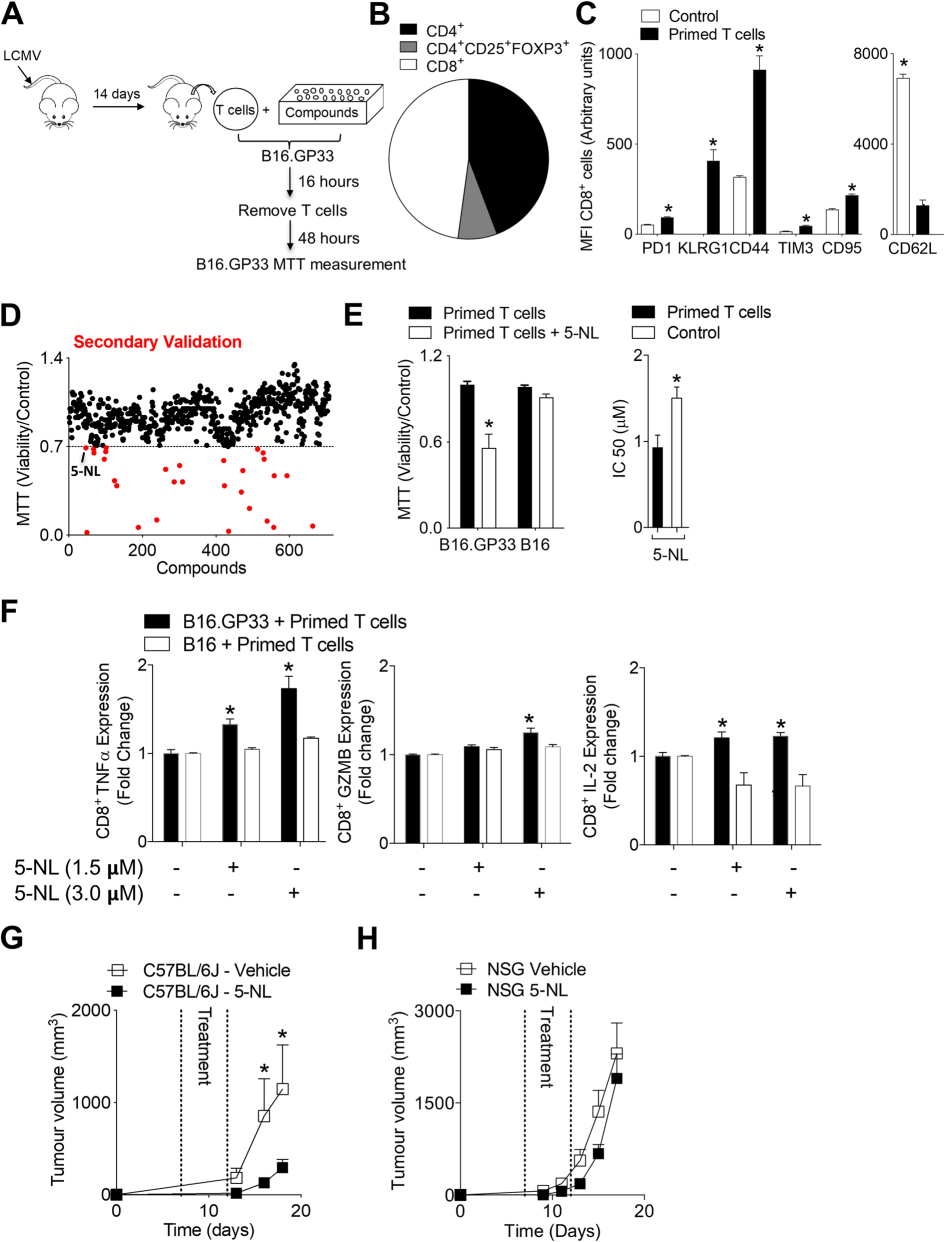
A pharmacological screen identifies the serotonin agonist 5-Nonyloxytryptamine (5-NL) as potentiating T cell mediated anti-tumor immunity. **A** Screen schematic is shown. **B**-**F** Mice were infected with 2 × 10^5^ pfu of LCMV-Armstrong. 14 days post infection, splenic pan-T cells were purified. **B** Splenic T cells were analyzed using FACS to obtain the ratio of CD8^+^, CD4^+^ and CD4^+^CD25^+^FoxP3^+^ T cells shown as percent composition (*n* = 3). **C** CD8^+^ T cells were evaluated for various surface markers using flow cytometry (*n* = 3). **D** Co-cultured LCMV-primed splenic pan-T cells and B16.GP33 cells were treated with 770 pharmacological compounds at a concentration of 1 μM. T cells were removed from co-culture at 16 h. Tumor-cell viability was assessed using the MTT assay 48 h post-treatment. Viability for each compound was expressed as a fraction relative to control (B16.GP33 cells + T cells). Potential hit compounds below the cut-off of 0.7 are shown in red. (E, Left Panel) B16.GP33 and B16 cells were co-incubated with LCMV-primed splenic pan-T cells with and without 5-NL (1 μM) as described in D and tumor cell viability was assessed using the MTT assay (*n* = 3–5). (E, Right Panel) B16.GP33 cells were co-incubated with LCMV-primed splenic pan-T cells with and without T-cells for 72 h and the IC50 was determined using the MTT assay (*n* = 4). **F** B16.GP33 and B16 murine melanoma cells were co-incubated with LCMV-primed splenic pan-T cells and 5-NL for 16 h. Intracellular staining of CD8^+^ T cells for TNFα, Granzyme B (GZMB) and IL-2 was measured using flow cytometry and normalized to its own respective cell line controls (*n* = 6). **G** C57BL/6 J or (**H**) NSG mice were subcutaneously injected with 5 × 10^5^ B16.GP33 cells. 7 days post-tumor injection, mice were randomized and into two groups and treated daily with 6.25 mg/kg of 5-NL or vehicle for five consecutive days and tumor volume was measured (*n* = 5–12). Error bars indicate SEM; *P < 0.05 as determined by a Student´s t-test (unpaired, 2 tailed), one or two-way ANOVA with a Dunnett’s post-hoc test

To identify novel drugs capable of modulating T cell anti-tumor immunity, we used the NIH Clinical Collection (NCC) composed of pharmacologically active small molecules. Splenic T cells were harvested from mice 14 days post infection with LCMV-Armstrong, a strain that causes robust effector CD8^+^ T cells responses and is rapidly cleared in wild-type mice following infection (Fig. 1A) [20–22]. Specific anti-B16.GP33 activity of pan purified T cells and CD8^+^ purified T cells was confirmed compared to B16.F10 parental controls (B16) by assessment of granzyme B (GZMB) and IL-2 production (Supplementary Fig. 1A). Since purified CD8^+^ T cells have an increased intrinsic response towards tumor cells expressing gp33 when compared to pan-T cells, the challenge to stimulate pan-T cells against tumor cells is greater in this setting and likely better recapitulates the complexity of the TME. Splenic pan-T cells from LCMV infected mice are composed of several T cell subsets including regulatory and naïve CD4^+^ T cells as well as CD8^+^ T cells (Fig. 1B). As expected, splenic CD8^+^ T cells were CD62L low compared to T cells from naïve mice and expressed killer cell lectin-like receptor subfamily G, member 1 (KLRG1) (Fig. 1C). Although residual levels of PD-1 and Tim-3 expression was detected in LCMV-primed T cells compared to naïve T cells, the low CD95 expression indicates that these T cells are mainly effector/effector memory T cells (Fig. 1C) [23]. To elicit anti-tumor activity, T cells were incubated with B16.GP33 cells (Fig. 1A). To uncover compounds capable of potentiating T cell activity, we titrated the T cell: target cell ratio to a T-cell sublethal anti-tumoral effect as assessed by the MTT assay (Supplementary Fig. 1B). Compounds were screened at a dose of 1 µM and those below a viability cut-off of 0.7 (70 percent viability relative to control cells + T cells) were considered potential hits (Fig. 1D and Supplementary Table 1). We reasoned that compounds affecting T cell immunity would result in decreased cancer cell viability in this setting and accordingly be identified as hits. As previously described, many compounds (mostly anti-chemotherapeutics or anti-metabolites) exhibited cytotoxic/anti-proliferative effects (IC50 < 250 nM, Supplementary Table 1) and these cytotoxic compounds were identified by comparing our results to a previous screen that used the same library to determine IC50 values in B16 melanoma cells [24]. Some potential hits (etoposide and docetaxel) are already known to modulate the immune system and T cells [25, 26]. A serotonin receptor (HTR) agonist 5-Nonyloxytryptamine (5-NL) was also a potential hit. Since another serotonin agonist, Tegaserod, modulated the tumor microenvironment (TME) by decreasing the infiltration of regulatory T cells (Tregs) [24], we opted to further validate 5-NL. Using the same ex vivo co-culture system as in the screen, 5-NL treatment resulted in significant decreased cancer cell viability of B16.GP33 but not B16 cells (Fig. 1E). Furthermore, addition of LCMV-primed effector T cells to a range of 5-NL doses resulted in a significantly lower half maximal inhibitory concentrations (IC50) values against B16.GP33 cells (Fig. 1E). When LCMV-primed T cells and tumor cells were co-incubated, 5-NL increased the expression of TNF alpha (TNFα), GZMB and IL-2 in CD8^+^ T cells incubated with B16.GP33 but not B16 cells (Fig. 1F).

Next, we wondered whether 5-NL had anti-tumoral effects in vivo. Using a syngeneic immune-competent model, B16.GP33 cells were subcutaneously inoculated into C57BL/6 J mice. Treatment with 5-NL, commencing when tumors became palpable, delayed tumor growth (Fig. 1G). We wanted to separate any potential 5-NL induced immune anti-tumoral effects from direct effects on tumor cells especially as 5-NL induced apoptosis in vitro at 72 h post 5-NL treatment (Supplementary Fig. 1C and D). We therefore subcutaneously inoculated the immunocompromised NSG mice with B16.GP33 cells and treated the mice with 5-NL. Treatment with 5-NL did not significantly alter tumor growth indicating that the immune system was crucial in mediating 5-NL’s anti-cancer effects in vivo (Fig. 1H). We next assessed tumors harvested from C57BL/6 J inoculated mice for markers of apoptosis using immunohistochemistry and images were scored using the IHC profiler [27]. At the early stage of tumor growth (day 13 post tumor inoculation), there were no significant differences of cleaved Caspase-3 and 8 between tumors harvested from 5-NL and vehicle treated mice (Supplementary Fig. 1E). Although active Caspase-9 was slightly increased in 5-NL tumors, differences were not apparent when assessed via immunoblot (Supplementary Fig. 1F). Taken together, we have uncovered a novel agent that has direct anti-tumor effects but is also capable of simultaneously boosting the immune system.

### 5-Nonyloxytryptamine (5-NL) improves T cell immunity in vivo but does not directly affect T cells

**Fig. 2.**
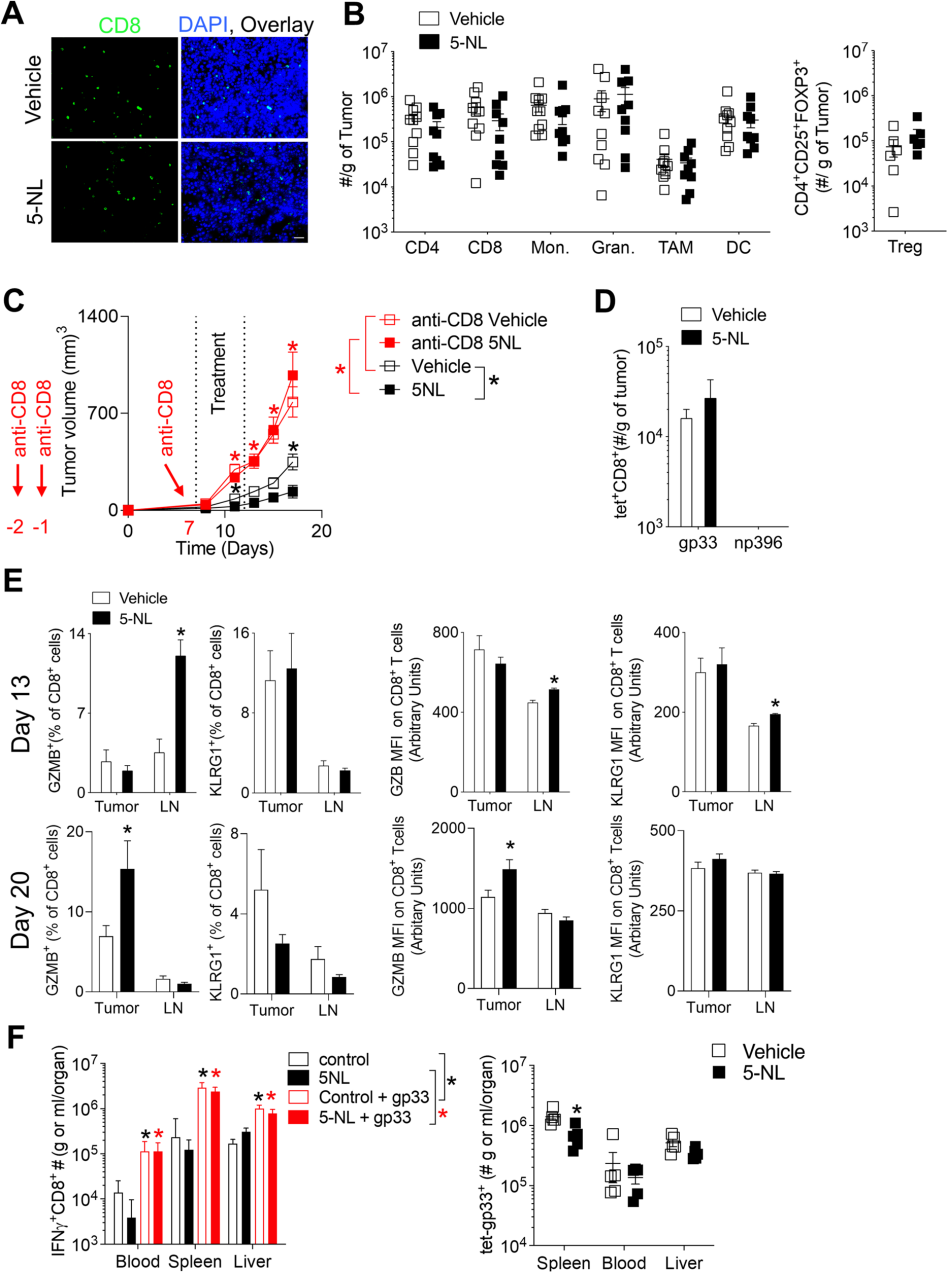
5-Nonyloxytryptamine (5-NL) improves T cell anti-tumor immunity in vivo. **A**-**E** C57BL/6 J mice were subcutaneously injected with 5 × 10^5^ B16.GP33 cells. 7 days post-tumor injection, mice were randomized into two groups and treated daily with 6.25 mg/kg of 5-NL or with vehicle for five consecutive days. Mice were sacrificed on 13 days post tumor-inoculation. **A** Tumor sections were stained for CD8^+^ T cells using immunofluorescence (a representative image of *n* = 4 is shown, scale bar indicates 50 µm). **B** Numbers of tumor infiltrating CD8^+^ and CD4^+^ T cells, Treg’s (CD4^+^CD25^+^FOXP3^+^), monocytes (CD11b^+^Ly6C^high^Ly6G^−^), granulocytes (CD11b^+^Ly6G^high^Ly6C^low^), tumor associated macrophages (TAMs, CD11b^+^F4/80^high^Ly6C^low^Ly6G^−^) and dendritic cells (DCs, CD11c^+^MHC-II^+^) were assessed using flow cytometry (*n* = 6–10). **C** In addition to the tumor inoculation and 5-NL treatment described in (A), C57BL/6 J mice were also treated with a CD8^+^ T cell depleting antibody (anti-CD8) on days -2, -1 and 7 pre and post tumor cell inoculation. Tumor volume was measured (*n* = 4–8). **D**-**E** Tumor and tumor-draining lymph node infiltrating CD8^+^ T cell markers, intracellular GZMB as well as tetramer were assessed by flow cytometry from mice sacrificed at day 13 (upper panel) or day 20 (bottom panel) post tumor inoculation (*n* = 5–11). **F** C57BL/6 J mice were infected with 2 × 10^5^ pfu of LCMV Armstrong and treated daily with 6.25 mg/kg of 5-NL or vehicle for 5 consecutive days starting at day 1 post-infection. 10 days post-infection, cells from the blood, spleen and liver were re-stimulated with LCMV-specific gp33 epitope followed by staining for IFNγ using FACS analysis (*n* = 5). Tet-gp33^+^ CD8^+^ T cells in the blood, spleen and liver were measured 10 days post-infection (*n* = 5). Error bars indicate SEM; *P < 0.05 as determined by a Student´s t-test (unpaired, 2 tailed), or a two-way ANOVA with a Tukey’s post-hoc test

Next, we wanted to investigate how 5-NL mechanistically improved anti-tumor responses in vivo. To characterize the TME, we harvested tumors at day 13 post inoculation when there were no differences in tumor size between vehicle and 5-NL treated groups (Fig. 1G). Tumors from vehicle and 5-NL treated mice showed CD8^+^ T cell infiltration (Fig. 2A). However, the number of CD8^+^ T cells did not alter following 5-NL treatment (Fig. 2B). Tumoral infiltration of other immune subsets including CD4^+^ T cells, monocytes (CD11b^+^Ly6C^high^Ly6G^−^), granulocytes (CD11b^+^Ly6G^high^Ly6C^low^), tumor-associated macrophages (TAMs, CD11b^+^F4/80^high^Ly6C^low^Ly6G^−^), dendritic cells (DCs, CD11c^+^MHC-II^+^) and regulatory T cells (Treg, CD4^+^CD25^+^FOXP3^+^) was also not changed by 5-NL treatment (Fig. 2B and Supplementary Fig. 2A and B). Although the infiltration of Treg’s was not different, the expression of the transcription factor GATA3 was lower in Treg’s harvested from tumors of 5-NL treated mice (Supplementary Fig. 2C).

Next, we tested the functional significance of the CD8^+^ T cell infiltrating subset in the context of 5-NL’s anti-tumoral activity. Upon depletion of CD8^+^ T cells (Supplementary Fig. 3A) tumor growth was increased relative to undepleted controls (Fig. 2C). This highlights the importance of CD8^+^ T cells in the tumor model (Fig. 2C). Notably, 5-NL’s tumor-suppressive phenotype was abrogated in the absence of CD8^+^ T cells indicating a dependency on CD8^+^ T cells for 5-NL-mediated anti-tumoral effects (Fig. 2C). Gp33 antigen specific CD8^+^ T cells were present in the tumor although there were no significant differences in tetramer-positive cell numbers between the 5-NL and vehicle treated groups (Fig. 2D). Another LCMV antigen, the H2-Db restricted np396 was used as a negative control (Fig. 2D). There was a higher percentage of GZMB producing CD8^+^ T cells and higher expression of KLRG1 which is upregulated in highly cytotoxic effector CD8^+^ T cells [28] in the tumor draining lymph node (LN) harvested from 5-NL treated mice at day 13 post tumor inoculation (Fig. 2E). When we assessed tumors and the tumor-draining lymph node from 5-NL and vehicle treated mice at day 20 post tumor inoculation, we observed higher cytotoxic CD8^+^ T cell effector activity (higher percentage and expression of GZMB producing CD8^+^ T cells) in the tumors of 5-NL treated mice (Fig. 2E).

Although 5-NL did not alter infiltration of various immune infiltrates including effector CD8^+^ T cells (Fig. 2B), TCR downregulation in the TME might underestimate the infiltration of antigen specific T cells as assessed by tetramer staining. We therefore transferred purified and activated splenic CD45.1^+^ CD8^+^ T cells from transgenic TCR (P14) mice recognizing the LCMV gp33 peptide [29] into tumor-bearing mice followed by vehicle or 5-NL treatment (Supplementary Fig. 3B). Most of the CD45.1^+^CD8^+^ cells homed into the tumor but their numbers and percentages were not different between 5-NL and vehicle treated mice (Supplementary Fig. 3C) corroborating the earlier finding that 5-NL did not alter infiltration or expansion of effector CD8^+^ T cells within the tumor.

Next we wondered whether 5-NL affected T cells intrinsically. 5-NL is a serotonin receptor (HTR) 1Dβ agonist (HTR1Dβ) that also has affinity for HTR1A-B, HTR2A and HTR2C [30]. There was a robust expression of HTRs in naïve T cells as well as tumor cells (Supplementary Fig. 4A and B respectively). Furthermore, members of Class 1 and 2 HTRs, including the ones targeted by 5-NL were upregulated in T cells following infection (Supplementary Fig. 4A). As serotonin signaling has been previously shown to be important for the activation of T cells [31–33], we reasoned that 5-NL might directly affect T cells through signaling of the HTRs to increase anti-tumor immunity. To test this, we treated LCMV-infected mice with 5-NL. We hypothesized that 5-NL might increase LCMV-triggered CD8^+^ T cell effector responses. However, 5-NL did not increase the frequency of tetramer gp33^+^ CD8^+^ T cell frequencies in mice infected with LCMV in the blood, spleen as well as liver and there were no differences between IFNγ, GZMB and TNFα positive CD8^+^ T cells in the blood, spleen and liver of LCMV infected mice following re-stimulation with gp33 (Fig. 2F and Supplementary Fig. 4C). Taken together, 5-NL does not improve T cell immunity in the context of acute viral infections. Therefore, its immunostimulatory effects are unlikely to occur through direct T-cell mediated effects. We confirmed this in our initial co-culture system by pre-treating the tumor cells with 5-NL, removing 5-NL, followed by incubation with LCMV-primed T cells. 5-NL pre-treatment increased the expression of Granzyme B (GZMB), TNF-α, IL-2 and surface activation marker KLRG1 in CD8^+^ T cells co-incubated with B16.GP33 cells (Supplementary Fig. 4D).

### 5-Nonyloxytryptamine (5-NL) upregulates antigen presenting machinery in vitro and in vivo without upregulating PD-L1

**Fig. 3:**
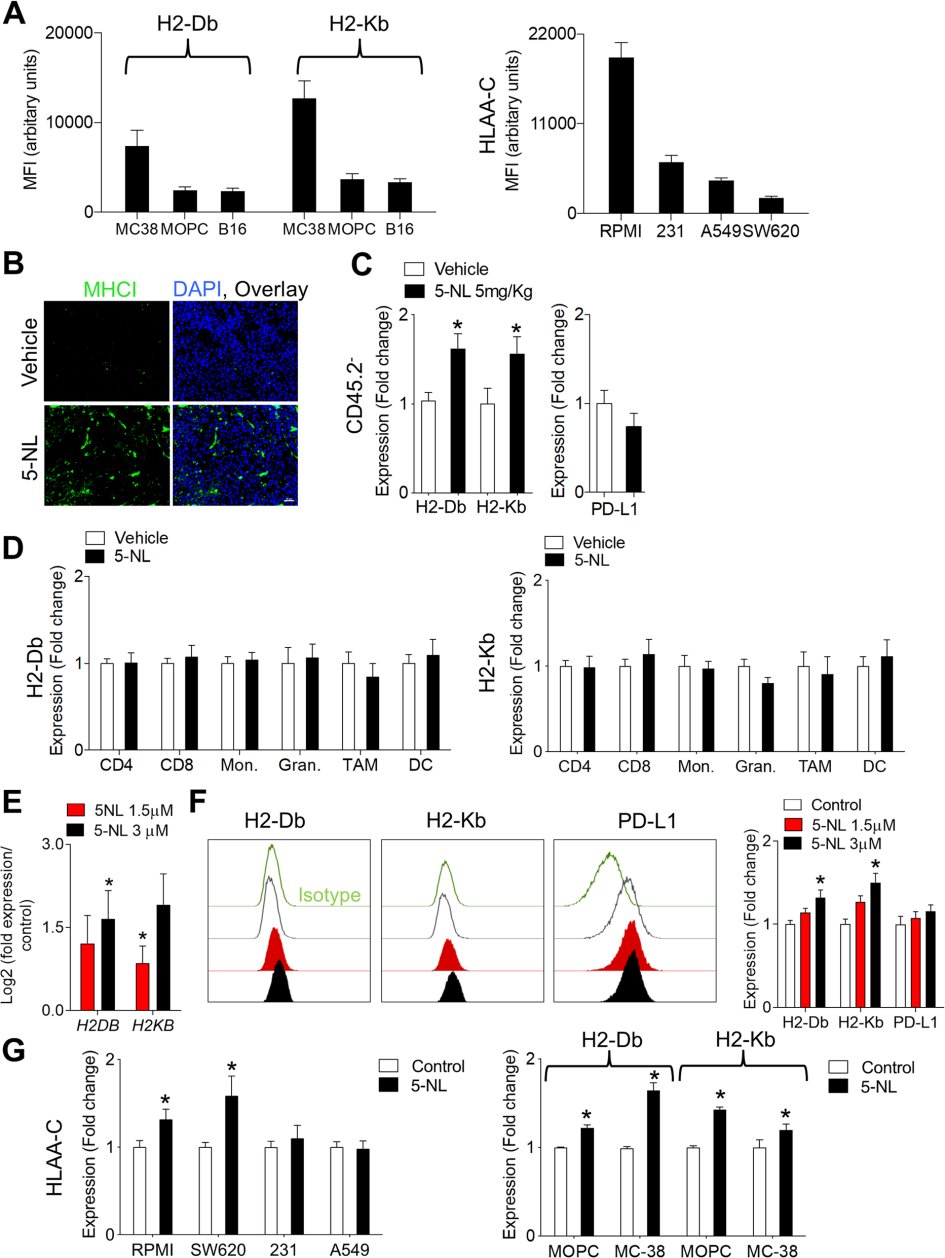
5-Nonyloxytryptamine (5-NL) upregulates antigen presenting machinery in human and murine tumors in vitro and in vivo*.* **A** Basal expression levels of H2-Db and H2-Kb in mouse cells and HLA A-C in human cells were assessed using flow cytometry (*n* = 5). **B**-**D** C57BL/6 J mice were subcutaneously injected with 5 × 10^5^ B16.GP33 cells. 7 days post-tumor injection mice were randomized into two groups and treated daily with 6.25 mg/kg of 5-NL or with vehicle for five consecutive days. Mice were sacrificed on day 13 post tumor-inoculation. **B** Tumor sections were stained for MHC-I using immunofluorescence (representative images of tumors harvested from 4 mice are shown; scale bar indicates 50 µm). **C** H2-Db/Kb and PD-L1 protein expression on CD45.2^−^ and (**D**) H2-Db/Kb expression on tumor infiltrating CD8^+^ and CD4^+^ T cells, Treg’s (CD4^+^CD25^+^FOXP3^+^), monocytes (CD11b^+^Ly6C^high^Ly6G^−^), granulocytes (CD11b^+^Ly6G^high^Ly6C^low^), TAMs (CD11b^+^F4/80^high^Ly6C^low^Ly6G^−^) and DCs (CD11c^+^MHC-II^+^) was assessed using flow cytometry (*n* = 5–16). **E** *H2DB* and *H2KB* mRNA expression in B16 cells treated with 5-NL for 18 h was assessed using RT-PCR. Expression was normalized to *β-Actin* (*n* = 4)*.* **F** H2-Db/Kb and PD-L1 protein expression was assessed by flow cytometry following treatment with 5-NL for 18 h in B16 cells (phenotype was recapitulated in B16.GP33 cells, data not shown) (*n* = 5). **G** H2-Db/Kb (mouse cell lines) and HLA A-C (human cell lines) protein expression was assessed using flow cytometry following treatment with 5-NL (5 μM for RPMI-7591 and 3 μM for MC-38, SW620, A549, MDA-MB-231 and MOPC cells) for 24 h (*n* = 5–9). Error bars indicate SEM; *P < 0.05 as determined by a Student´s t-test (unpaired, 2 tailed) or a one-way ANOVA with a Tukey’s post-hoc test

As 5-NL did not improve T cell immunity in a tumor-free infection model, we postulated that 5-NL might affect the TME to promote anti-tumor immunity. B16 MHC-I expression (as assessed by measuring the H2-Db and H2-Kb isoforms comprising the MHC-I complex in C57BL/6 mice [34]) is low (though detectable as also previously reported [35]), relative to other murine and human cell lines including the immunogenic MC-38 cells (Fig. 3A). We therefore wondered whether 5-NL affected MHC-I expression in tumor cells.

When we stained for MHC-I whose relative low expression in part defines an immunologically ignorant phenotype [36, 37], we observed that tumors harvested from 5-NL treated mice had significantly higher expression of MHC-I (Fig. 3B). Next, we checked the expression of MHC-I molecules H2-Db and H2-Kb on tumor and tumor infiltrating immune cells using FACS analysis. To differentiate between tumor cells and immune infiltrating leukocytes, we used CD45.2 as a tumor infiltrating leukocyte (TIL) marker and found that both H2-Db and H2-Kb were significantly upregulated on CD45.2^−^ cells harvested from 5-NL treated mice but not in tumor infiltrating lymphocytes (Fig. 3C-D and Supplementary Fig. 5A). As MHC-I shares some transcriptional elements with MHC-II such as the SXY regulatory module [38], we wondered whether 5-NL treatment also impacted MHC-II expression. However, MHC-II expression was not changed in immune infiltrates or CD45.2^−^ cells in the tumors of 5-NL treated mice compared to controls (Supplementary Fig. 5B). MHC-I upregulation is often accompanied by PD-L1 upregulation [39] but 5-NL treatment did not result in concomitant increased PD-L1 expression in CD45.2^−^ cells (Fig. 3C). Taken together, 5-NL upregulated MHC-I molecules H2-Db and H2-Kb in CD45.2^−^ cells within the TME in vivo and we therefore hypothesized that 5-NL improved anti-tumor immunity through upregulation of H2-Db and H2-Kb in tumor cells. Indeed, 5-NL induced the expression of H2-Db and H2-Kb in B16 cells 18 h post 5-NL treatment without affecting PD-L1 (Fig. 3E and F). Expression of other members of the antigen presenting machinery including *B2M* were also increased in response to 5-NL treatment in melanoma cells (Supplementary Fig. 5C). To ensure that the upregulation of MHC-I antigen presenting machinery was not merely a consequence of apoptosis induction (evident at 72 h post 5-NL treatment, Supplementary Fig. 1 C-D), we treated melanoma cells with another serotonin agonist known to induce melanoma tumor cell apoptosis, Tegaserod (TM) [24]. Treatment with TM did not lead to upregulation of H2-Db and H2-Kb (Supplementary Fig. 5D).

Next, we wondered about the susceptibility of human cell lines to 5-NL induced MHC-I upregulation. Upon treatment with 5-NL, HLA A-C was upregulated in human melanoma RPMI-7591 (RPMI) and colon SW620 cells but not in human breast and lung adenocarcinoma cells (Fig. 3G). H2-Db and H2-Kb expression was increased in murine colon MC-38 cells and squamous oropharynx carcinoma MOPC cells (Fig. 3G). MHC-II was not upregulated in most cells following 5-NL treatment (Supplementary Fig. 5E). In the MC-38 as in B16.GP33 cells (Fig. 3B), there was also strong MHC-I upregulation in vivo (Supplementary Fig. 5F). B16 melanoma cells are poorly immunogenic and this has been attributed to relative low MHC-I expression [35]. Expression of HLA A-C also varied across the human cell lines but 5-NL was able to upregulate MHC-I/HLA A-C expression in cell lines of varying immunogenicity (Fig. 3G).

### 5-Nonyloxytryptamine (5-NL) and other inducers of CREB phosphorylation recapitulate MHC-I upregulation independent of IFNγ signaling

**Fig. 4:**
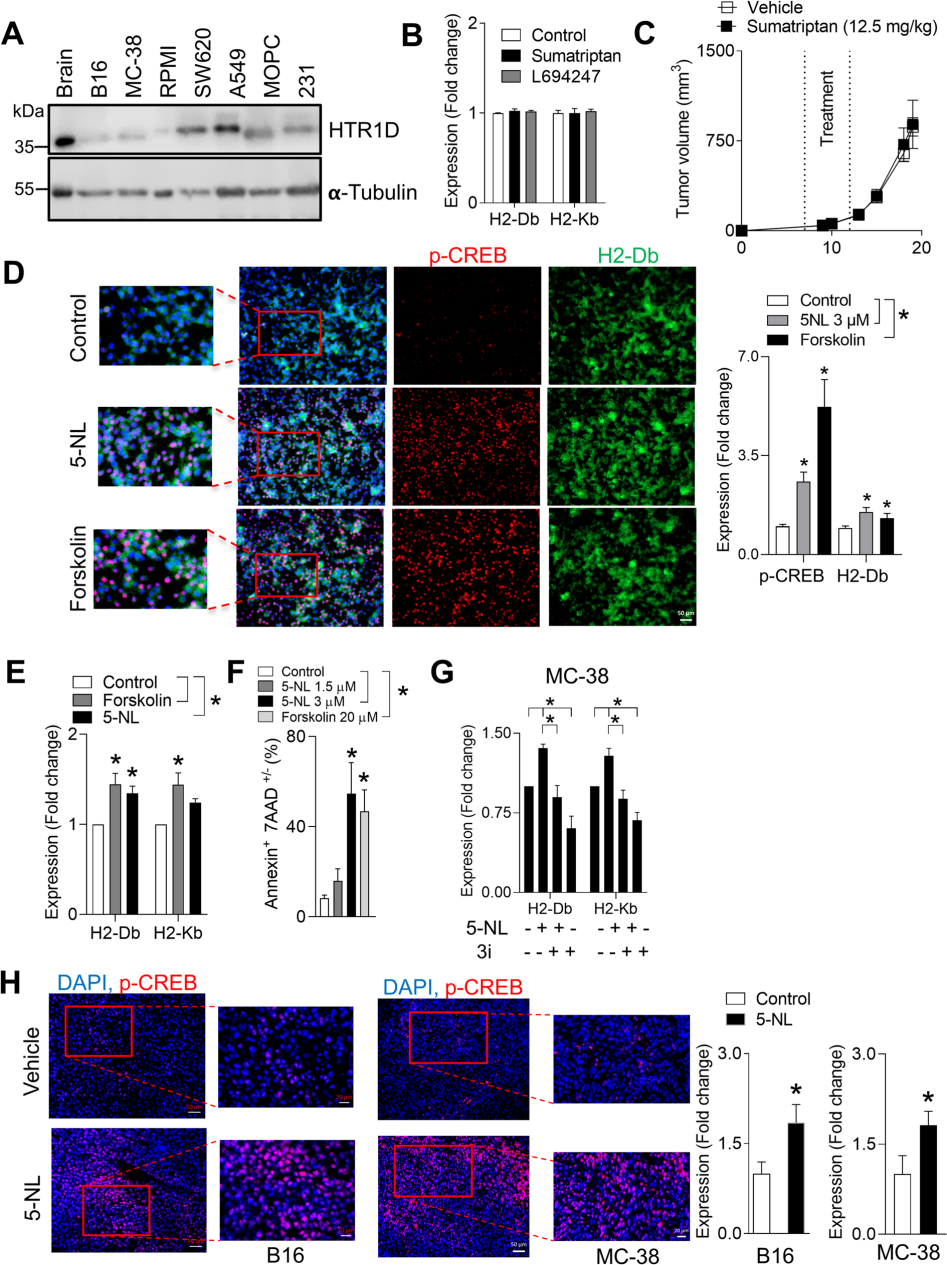
5-Nonyloxytryptamine (5-NL) and other inducers of CREB activation upregulate antigen presenting machinery in vitro and in vivo*.* **A** Protein expression of HTR1D in cancer cell lines was assessed using immunoblot analysis (a representative immunoblot of *n* = 3 is shown, cropping is indicated by a black frame). Lysates harvested from the mouse brain were used as a positive control. **B** Treatment with other HTR1D agonists for 18 h (Sumatriptan and L694247, both 3 μM) did not increase H2-Db/Kb protein expression in B16 cells as assessed using FACS (*n* = 3). **C** C57BL/6 J mice were subcutaneously injected with 5 × 10^5^ B16.GP33 cells. 7 days post-tumor injection mice were randomized into two groups and treated daily with 12.5 mg/kg of Sumatriptan or with vehicle for five consecutive days. Tumor volume was measured (*n* = 6–7). (**D**, left panel) Representative immunofluorescent images of B16 cells treated with 5-NL (3 µM) or forskolin (10 µM) for 18 h and stained for phosphorylated CREB (p-CREB Ser-133) and H2-Db are shown (representative images of *n* = 3–4 are shown; scale bar indicates 50 µm) and fluorescent signal is quantified in D, right panel. **E** The adenylyl cyclase activator forskolin (20 μM) increased H2-Db/Kb protein expression in B16 cells following 18 h of treatment (*n* = 6) as measured by FACS. **F** B16 cells were treated with 5-NL and forskolin at the indicated doses for 72 h. Apoptosis was assessed using Annexin V/7AAD staining (*n* = 5). Percent apoptosis was ascertained by summing up the Annexin V^+^/7AAD^−^ and Annexin V^+^/7AAD^+^ populations. **G** MC-38 cells were pre-treated for 30 min with the p-CREB inhibitor 3i (8 μM) followed by treatment with 3 µM of 5-NL for 24 h. Cells were analyzed using FACS for expression of H2-Db /Kb (*n* = 3–5). **H** C57BL/6 J mice were subcutaneously injected with 5 × 10^5^ B16.GP33 or MC-38 cells. 7 days post-tumor injection mice were randomized into two groups and treated daily with 6.25 mg/kg of 5-NL or with vehicle for five consecutive days. Mice were sacrificed on 13 days post tumor-inoculation and tumor tissue was stained for p-CREB using immunofluorescence. Scale bars indicate 50 µm and 20 µm (region of interest outlined in red) (representative images of tumors harvested from 4–6 mice are shown). Fluorescent signal from p-CREB channel was quantified in the right panel. Error bars indicate SEM; *P < 0.05 as determined by a Student´s t-test (unpaired, 2 tailed) or a one-way ANOVA with a with a with a Dunnett’s post-hoc test

5-NL was designed to be an HTR1Dβ agonist. Although protein expression of HTR1D was confirmed in multiple cell lines (Fig. 4A), treatment with other HTR1Dβ agonists Sumatriptan and L694247 failed to recapitulate the H2-Db and H2-Kb upregulation (Fig. 4B). Consistently, dosing with the FDA approved Sumatriptan failed to delay tumor growth in vivo (Fig. 4C). Furthermore, knock-down with esiRNA’s targeting HTR1D or knock-out of HTR1D using CRISPR-Cas9 did not alter 5-NL’s ability to upregulate H2-Db (Supplementary Fig. 5G and H). Additionally, knock-down with an esiRNA targeting HTR2A, another putative 5-NL target, did not alter 5-NL’s ability to upregulate H2-Db (Supplementary Fig. 5I). Treatment with serotonin did not impact H2-Db expression and co-treatment of 5-NL and the pan serotonin receptor inhibitor, Asenapine did not alter 5-NL’s ability to upregulate H2-Db (Supplementary Fig. 5 J). Taken together, we surmise that 5-NL mediated improved anti-tumor immunity occurred irrespective of signaling through receptors HTR1Dβ and HTR2A. 5-NL’s affinity for other receptors including HTR1A-B and HTR2C [30] broadens the range of targets since human melanoma cells do express other HTR’s [24] as do murine B16 and MC-38 cells (Supplementary Fig. 4B). However, as treatment with serotonin and co-treatment of 5-NL and Asenapine did not recapitulate or block H2-Db upregulation respectively, this makes involvement of other HTRs less likely.

In order to uncover signaling pathways responsible for 5-NL’s upregulation of antigen presenting machinery, we further immunoblotted transcription factors known to be involved in canonical serotonin signaling and in MHC-I gene transcription. Interferon γ (IFNγ) is a potent transcriptional inducer of MHC-I expression [40, 41]. However, there were no differences in IFNγ protein levels within the tumors of 5-NL or vehicle treated mice (Supplementary Fig. 6A) or mRNA levels of IFNγ and type I interferons (Supplementary Fig. 6B). Consistently*,* 5-NL treatment of B16 cells did not induce the expression of IFNγ and type I interferons (Supplementary Fig. 6C). Moreover, while exogenous treatment with IFNγ robustly increased STAT1 levels in B16 cells, treatment with 5-NL did not (Supplementary Fig. 6D). However, treatment with IFNγ upregulated MHC-I/HLAA-C in every cell line except the MOPC cells (Supplementary Fig. 6E). As expected, the upregulation was strongest in cells with low basal levels of MHC-I/HLAA-C such as B16 cells. This was accompanied by concomitant PD-L1 upregulation which again, 5-NL failed to upregulate in every cell line tested (Supplementary Fig. 6F and Supplementary Table 2). Taken together, we concluded that 5-NL-induced MHC-I upregulation independently from IFNγ.

NF-κB can regulate MHC-I genes through binding to their enhancers [41]. Phosphorylated NF-κB p100 was not detectable in B16 and MC-38 cells and treatment with 5-NL did not alter total and phosphorylated NF-κB p65 levels (Supplementary Fig. 7A and B). Mitogenic pathways potentially influenced by serotonin signaling such as MAPK were unaffected by 5-NL treatment, although a decrease in the activation of the PI3K/Akt/mTOR pathway (decreased p70 S6 phosphorylation) was observed in B16 cells (Supplementary Fig. 7C). cAMP response element binding protein (CREB) has been shown to bind to MHC-I promoters [40–42], and activation of Gα_s_-coupled receptors (HTR4-7) occurs through protein kinase A (PKA) mediated phosphorylation of CREB (p-CREB) [43].

A robust increase in phosphorylation of CREB following treatment with 5-NL was observed in both B16 and MC-38 cells (Fig. 4D and Supplementary Fig. 8A and B). Notably, colorectal and melanoma cancer cells both expressed basal levels of p-CREB, which is consistent with what is reported in the literature [44]. Next, we investigated whether the changes in CREB phosphorylation were linked to the upregulation of antigen presenting machinery. Knockdown approaches of CREB are difficult in cancer cell lines as it often leads to cell death [45, 46]. Indeed, transient knockdown using esiRNA approaches in both cell lines led to an induction of apoptosis especially in B16 cells (Supplementary Fig. 8C). Using pharmacological inhibitors and activators to further dissect the mechanism of MHC-I upregulation in B16 and MC-38 cells, we observed a robust CREB phosphorylation and upregulation of H2-Db and H2-Kb upon treatment with the adenylate cyclase (AC) activator forskolin which is upstream of p-CREB [43] (Fig. 4D, E and Supplementary Fig. 8A, E). Forskolin also induced apoptosis in B16 and MC-38 cells although to a lesser extent than 5-NL in MC-38 cells (Fig. 4F and Supplementary Fig. 8F). Co-treatment of forskolin with 3i, a CREB inhibitor [47], blunted the forskolin-mediated H2-Db/Kb upregulation in MC-38 cells (Supplementary Fig. 8D). Importantly, co-treatment of 5-NL and 3i abolished 5-NL mediated H2-Db and H2-Kb upregulation in MC-38 cells (Fig. 4G). The sensitivity of B16 cells to CREB inhibition resulted in significant induction of apoptosis following short-term treatment with the 3i inhibitor. This would confound any interpretation of MHC-I upregulation thereby precluding co-treatment of 5-NL and 3i (Supplementary Fig. 8G). Notably, treatment of MC-38 cells with the p-CREB 3i inhibitor alone resulted in slightly decreased H2-Db levels (Supplementary Fig. 8D,G). Consistent with the in vitro results, p-CREB was increased in tumors from 5-NL treated mice in both melanoma and colon cancer in vivo (Fig. 4H). Taken together, as shown by pharmacological manipulation, the 5-NL mediated upregulation of the antigen presenting machinery is linked to increases in CREB phosphorylation.

### 5-Nonyloxytryptamine (5-NL) induces differential gene expression in tumor cells and activates the AMPK pathway

**Fig. 5:**
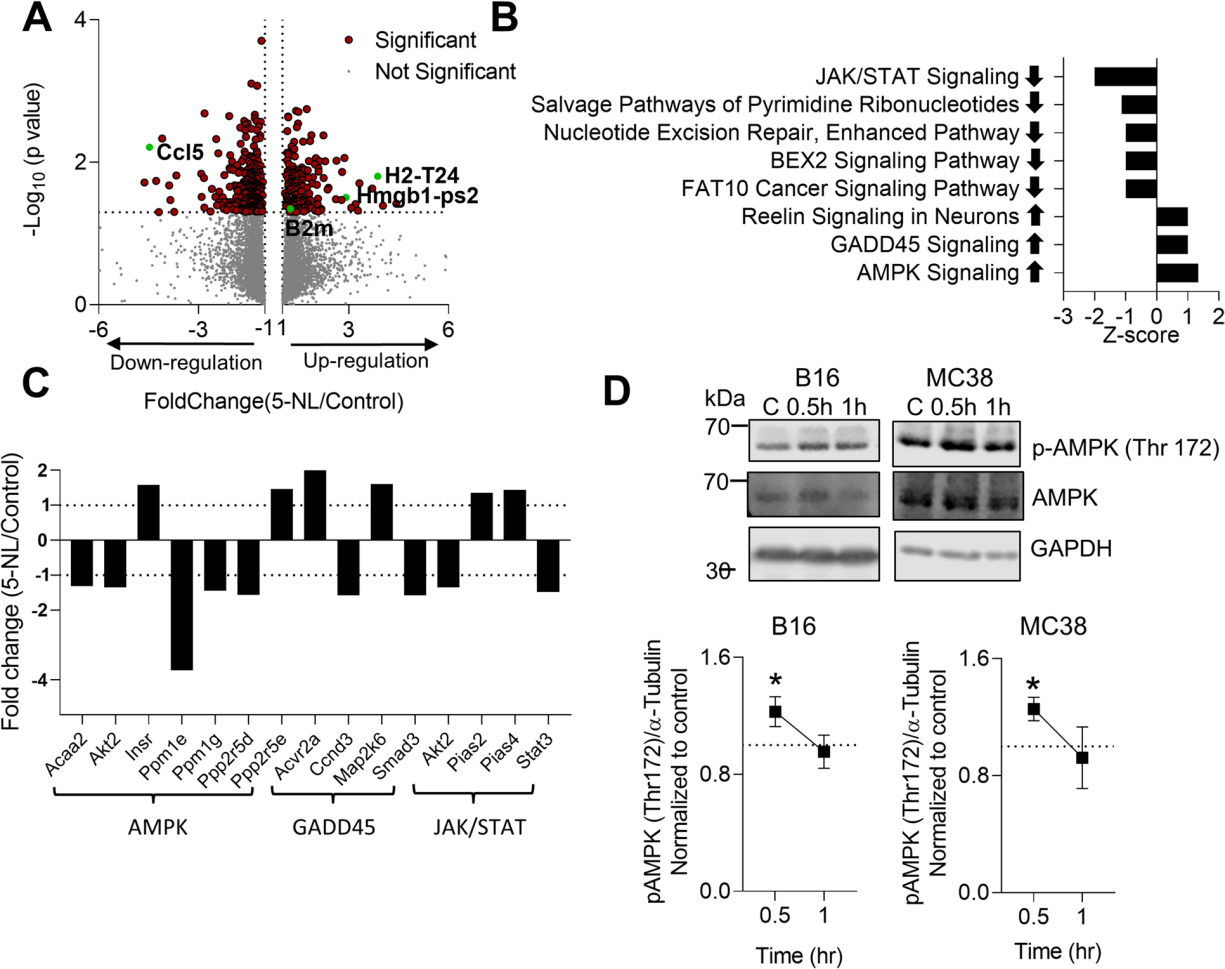
5-Nonyloxytryptamine (5-NL) induces differential gene expression and activates the AMPK pathway in tumor cells*.* B16.GP33 cells were treated with 5-NL (3 µM) for 18 h and RNA was assessed using RNA-seq analysis. **A** A Volcano Plot of the fold change gene distribution is shown. **B**-**C** GSEA analysis of pathways altered in 5-NL treated B16.GP33 cells is shown with arrow pointing up indicating pathway activation and arrow pointing down indicating downregulation. Fold changes in individual genes in select pathways are shown in C (*n* = 4). (D) Level of AMPK phosphorylation (Thr172) was assessed using immunoblot analysis in B16 and MC38 cells treated with 3 µM of 5-NL at the indicated time points quantified in the right panel (black frames indicate cropped immunoblot; *n* = 7–9). Error bars indicate SEM; *P < 0.05 as determined by a one-way ANOVA with a Dunnett’s post-hoc test

To determine what other signalling pathways were perturbed by 5-NL, we performed RNA-seq analysis on B16.GP33 cells. As CREB phosphorylation occurred at or after 18 h post-treatment, the RNA-Seq was performed at 18 h to determine any potential earlier causative 5-NL-induced perturbations. There were 468 genes differentially expressed between the 5-NL and control groups (Supplementary Table 3). In addition to *B2M,* an MHC-I gene involved in antigen processing, binding and presentation *H2-T24* was highly upregulated following 5-NL treatment (Fig. 5A). *H2-T24* is an MHC-Ib gene that has been shown to be significantly expressed in adult spleen and thymus tissues of C57BL/6 mice [34]. Interestingly, High Mobility Group Box 1 pseudogene 2 (*Hmgb1-ps2)* was amongst the highly upregulated genes in 5-NL treated B16.GP33 cells. Hmgb1 and its pseudogenes are multifunctional redox sensitive proteins that play an important role in anti-tumoral immunity. Hmgb1 is a damage-associated molecular pattern (DAMP) and its release by tumor cells facilitates immunogenic cell death (ICD) [48]. GSEA on the differentially expressed genes was implemented to determine prominent pathways altered between control and 5-NL treated B16.GP33 cells (Fig. 5B and C). Notably, the AMPK pathway was activated in 5-NL treated B16.GP33 cells. We verified that AMPK was activated in both B16 and MC-38 cells following 5-NL treatment as early as 30 min post-treatment (Fig. 5D and Supplementary Fig. 9A). Taken together, using RNA-Seq analysis we have not only confirmed our previous findings involving upregulation of antigen presenting machinery but have identified that 5-NL affects AMPK activation.

### 5-Nonyloxytryptamine (5-NL) can be successfully combined with immunotherapy in vivo

**Fig. 6:**
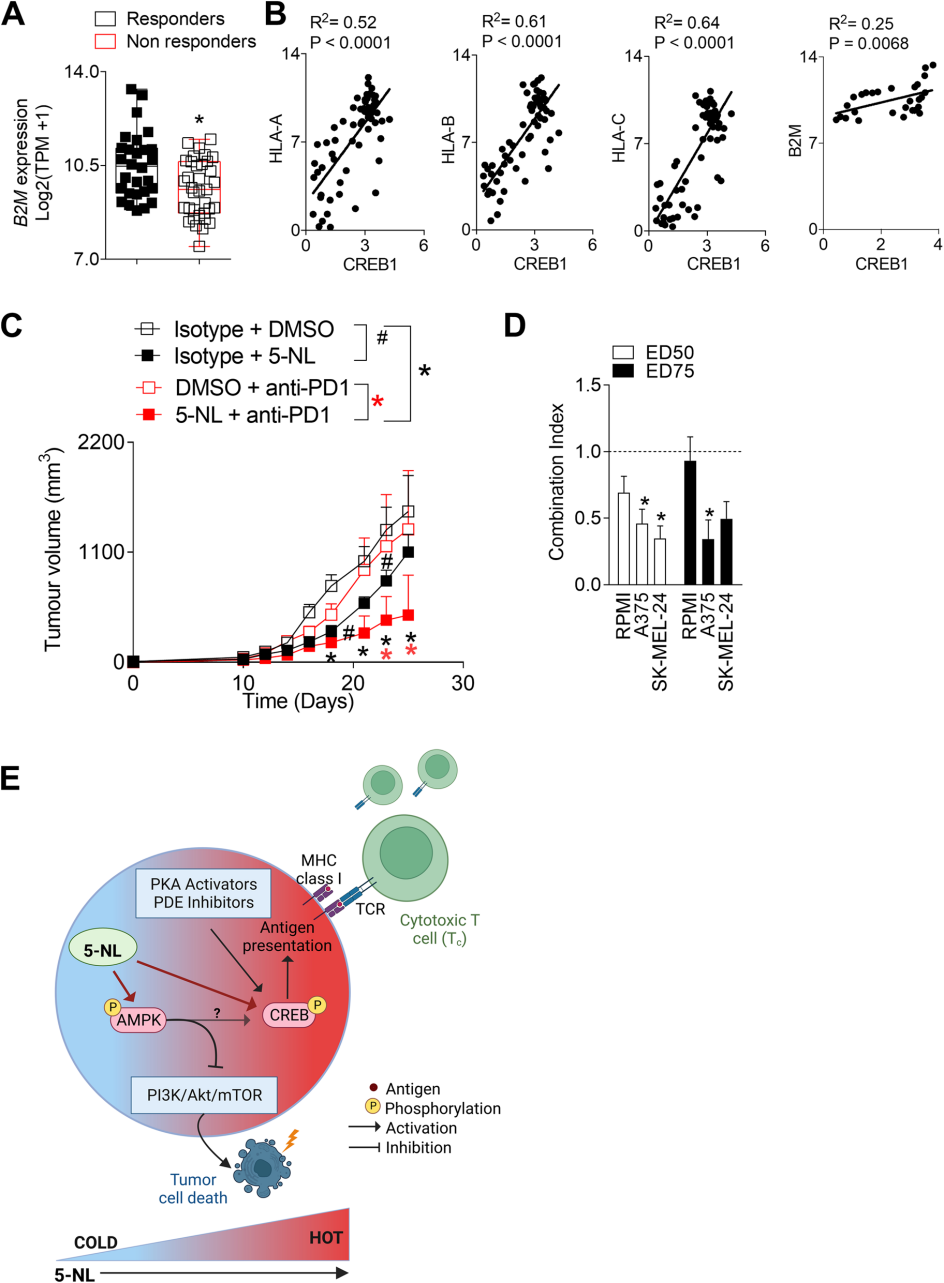
5-Nonyloxytryptamine (5-NL) can be successfully combined with immunotherapy in vivo*.* Transcriptomic data from melanoma samples of therapy naïve patients was mined from the Cancer Immunome Atlas. **A** Responders to checkpoint inhibitors (anti-PD1 and anti-CTL4) expressed higher mRNA levels of *B2M*. **B** Expression of *CREB1* positively correlated with *HLA A-C* and *B2M.* **C** C57BL/6 J mice were subcutaneously injected with 5 × 10^5^ B16.GP33 cells. 7 days post-tumor injection mice were randomized and treated daily with 6.25 mg/kg of 5-NL or with vehicle for five consecutive days. Additionally, mice were intravenously injected with murine anti-PD1 antibody or isotype control on days -1, 1, 3, 5 and 7 pre and post tumor inoculation. Tumor volume was measured (*n* = 5–9). Data is pooled from two independent in vivo experiments. **D** Combination index (CI) was calculated from dose response curves of human melanoma cell lines treated with 5-NL, Vemurafenib or in a combination in ratio 1:1. CI < 1 indicates synergy, CI = 1 indicates additivity, and CI > 1 indicates antagonism. The EC50 (50% effective concentration) and EC75 (75% effective concentration) are shown (*n* = 3). Error bars indicate SEM; *P < 0.05 as determined by a Student´s t-test (unpaired, 2 tailed) or a two-way ANOVA with a Tukey’s post-hoc test. **E** A schematic diagram summarizing 5-NL’s anti-tumoral and pro-immune effects is shown. The diagram created with BioRender.com

We used two different studies to mine transcriptomic data from the Cancer Immunome Atlas (TCIA) [49]. Van Allen et al. correlated genomic and transcriptomic data with response to CTLA4 blockade [50] while Hugo et al. with response to anti-PD1 therapy [51], both using metastatic melanoma samples collected prior to immunotherapy treatment. As also reported by Van Allen et al., there was no correlation between expression of HLAA-C and clinical benefit with anti-CTLA4 therapy. Combined transcriptomic data from both studies showed that immunotherapy responders had significantly higher expression of *B2M* than immunotherapy non-responders (Fig. 6A). Furthermore, strong and significant correlations were observed between *CREB1* expression and *HLAA-C* as well as *B2M* (Fig. 6B). Although expression of *HLAA-C* as well as *B2M* did not correlate strongly with *IFNG,* there was also a strong correlation between the IFNγ responsive gene *IFIT3* suggesting that it is difficult to ascertain the specific causes of these correlations (Supplementary Fig. 9B). Accordingly, we wondered whether 5-NL would impact response to checkpoint inhibition. Not surprisingly, due to the low immunogenicity of the B16.GP33 melanoma cell line, treatment with the anti-PD1 antibody had little effect as a single agent. Although tumors grew slower in the 5-NL/Isotype group compared to Vehicle/Isotype, tumor growth in the 5-NL/anti-PD1 group was significantly delayed when compared to the Vehicle/anti-PD1 group and the Vehicle/Isotype group (Fig. 6C). Additionally, we wanted to assess whether 5-NL could be potentially used in combination therapy with the standard of care treatment for late-stage melanoma B-Raf inhibitor Vemurafenib [52]. We ascertained the combination index following treatment with 5-NL and Vemurafenib on human melanoma cell lines RPMI, A375 and the SK-MEL-24, harboring the BRAF^V600E^ mutation and found a synergistic effect in A375 and SK-MEL-23 cell lines (Fig. 6D). Taken together, we therefore propose a model whereby 5-NL could increase the immunogenic profile of tumors and be combined with immunotherapies (Fig. 6E).

## Discussion

5-NL increased T cell anti-tumor immunity through up-regulation of the antigen presenting machinery in tumor cells while simultaneously inducing tumor cell apoptosis. 5-NL was developed as an HTR1Dβ agonist. However, 5-NL also has affinity for other HTR receptors also expressed by tumor cells in our system namely HTR1A-B, HTR2A and HTR2C [30]. HTR1A-F signaling occurs through G protein-coupled receptors (GPCRs) with inhibitory effects on adenylyl cyclase and corresponding decreased production of cAMP [43]. We observed increases in p-CREB following 5-NL treatment and other HTR1D agonists had no effects on MHC-I upregulation. This, combined with the fact that HTRID knockout and knockdown did not abrogate the phenotype, makes it is likely that 5-NL’s effects are independent of HTR1 signaling. HTR2A-C signaling occurs through activation of Gα_q_, PI3K/AKT, and ERK 1/2 pathways. There were no differences in ERK phosphorylation and the PI3K/AKT was blunted following 5-NL treatment. The mechanism of action could occur through canonical serotonin receptor signaling involving other HTR’s [4, 6, 7] whose downstream signaling involves the cAMP/PKA/CREB pathway [53]. However, there is no reported binding activity of 5-NL to HTR4,6–7. Likely the observed effects are rather mediated by the earlier pathways perturbed by 5-NL particularly by the AMPK pathway which has been shown to activate CREB [54]. The activation of cAMP/PKA/CREB pathway in our system is plausibly responsible for the increases of antigen presenting machinery given that the MHC-I upregulation phenotype was recapitulated by the AC activator forskolin and 5-NL’s action inhibited when combined with a p-CREB inhibitor. Recently, it was demonstrated that decreased AMPK activity in tumor cells led to attenuated antigen presentation and favoured an immunosuppressive TME [55]. Thus one can speculate that 5-NL-mediated early AMPK activation affects p-CREB and subsequently the antigen presenting machinery. However, AMPK activation has also been demonstrated to inhibit ribosomal protein p70 S6 kinase [56], which we also observe in our system following 5-NL treatment. As inhibition of PI3K/Akt/mTOR could be responsible for 5-NL’s cell death-inducing effects as has been shown in similar systems [24, 57], it is not clear whether AMPK activation might be responsible for 5-NL induced MHC-I upregulation (upstream of p-CREB) or 5-NL induced cell death (upstream of PI3K/Akt/mTOR). The upregulation of MHC-I is probably uncoupled from the apoptosis inducing effects of 5-NL as evidenced by the modest increases in forskolin-mediated apoptosis at least in MC-38 cells. Consistently, our data indicate that inhibition of p-CREB can prevent upregulation of MHC-I following 5-NL treatment in tumor cells.

Clinically, tumors have variable MHC-I expression that tends to be suppressed in later stages of progression making tumors refractory to checkpoint inhibition [58–61]. Therefore, to elicit responsiveness to immunotherapies, the conversion of poorly inflamed cold tumors into hot tumors such as through MHC-I upregulation [16] is therapeutically attractive as it might stimulate anti-tumor T cell immunity not only in the tumor tissue but also in the LN as was observed in our system. Notably, we observed this in our study with the B16 cells that have a reversible MHC-I deficient phenotype and intrinsic resistance to immunotherapy [35]. Treatment with the anti-PD1 antibody alone had no effect on tumor growth but the combination of 5-NL and anti-PD1 was most effective. Current clinical efforts of administering systemic IFNγ to increase MHC-I expression [62] are fraught with challenges namely rate-limiting toxicity, PD-L1 upregulation and T cell dysfunction [63–65]. Furthermore, as a means of primary and acquired resistance, many tumors inactivate interferon signaling [66]. In our study, MHC-I/HLAA-C was upregulated following IFNγ treatment in tumor cells of varying basal MHC-I/HLAA-C expression. This was accompanied by strong PD-L1 upregulation. While the effects of 5-NL mediated MHC-I/HLAA-C upregulation were less dramatic than with IFNγ, there was also no PD-L1 upregulation observed in vitro and in vivo*,* precluding 5-NL involvement in IFNγ/STAT1 signaling. Furthermore, 5-NL upregulated MHC-I expression in MOPC cells which were refractive to IFNγ-mediated MHC-I upregulation. This broadens its applicability and demonstrates that it can be used to boost responses across a wide tumor immunogenicity range.

In vivo*,* 5-NL was well tolerated but the clinical applicability of 5-NL remains to be determined as it is not FDA approved. There is the possibility of using other already FDA-approved compounds that also increase MHC-I through cAMP/PKA/CREB mediated signaling. For example, phosphodiesterase (PDE) inhibitors are drugs that elevate cAMP/cGMP levels and often activate CREB. They are already being evaluated as re-purposed anti-cancer agents and may be of clinical benefit [67–69]. The possibility of exploring the combination of already FDA-approved PDE inhibitors with checkpoint inhibitors to augment their efficacy is subject to future investigations.

## Conclusions

This study demonstrates novel therapeutic opportunities for the augmentation of immunotherapies. MHC-I upregulation in tumor cells using 5-Nonyloxytryptamine was uncoupled from increases in PD-L1 expression and modulated through CREB signaling. Increased antigen expression converted poorly immunogenic ‘cold’ tumors into ‘hot’ and elicited responsiveness to anti-PD1 immunotherapies.

## Materials and methods

### Cell culture and compounds

B16.F10 and B16.gp33 (Kindly provided by Dr. H.P. Pircher), MC-38, A549, MDA-MB-231 cell lines were maintained Dulbecco Modified Eagle's Medium (DMEM). Human RPMI-7951 cells were maintained in Eagle’s MEM. MOPC cells were cultured as previously described [70]. Media was supplemented with 10% FCS (15% for SK-MEL24) and penicillin/streptomycin. Cells were incubated at 37** °C** in 5% CO2, and routinely confirmed to be mycoplasma-free (MycoAlert Mycoplasma Detection Kit, Lonza). For screening, the NIH Clinical Collection (NCC) composed of 770 small molecules was used in the screen. 5-Nonyloxytryptamine, Sumatriptan, Forskolin (all from Sigma), Asenapine and the p-CREB 3i inhibitor (SelleckChem) were dissolved in DMSO. Serotonin was dissolved in water (SelleckChem).

### EsiRNA and CRISPR-Cas9

Cells were seeded and 24 h later, transfected with esiCREB (EMU067571, Sigma), esiHTR1D (EMU075541, Sigma), esiHTR2A (EMU023531, Sigma) or control esiFluc, (EHUFLUC, Sigma) using Lipofectamine 3000 (ThermoFisher) according to the manufacturer’s instructions. Lentivirus carrying plasmid pGk1.2Cas9 overexpressing Cas-9 protein, plasmid LRGFP2.1 overexpressing GFP protein with cloned sgRNA (sgHTR1D1, sequence TGTACTGCCCACTGCTAGTT) or LRCherry2.1 plasmid overexpressing mCherry protein with cloned sgRNA2 (sgHTR1D2, sequence CTGGCTAACAATGGTACGGA) were produced using Lenti-X Packaging Single Shots (VSV-G) (Takara Bio) in Lenti-X 293 T cells according to the manufacturer’s instructions. Cells were firstly transduced with pGk1.2Cas9 lentivirus and selected with puromycin for Cas-9 overexpressing clones. Next, cells were transduced with lentivirus carrying LRGFP2.1-sgHTR1D1 and LRCherry2.1-sgHTR1D2 simultaneously and sorted for double positive cells followed by clonal selection.

### Viruses

LCMV Armstrong (kindly provided by Rolf Zinkernagel, University of Zurich, Zurich, Switzerland) was propagated in L929 cells as previously described [71].

### T cell isolation

Splenic T cells were harvested from the spleens using the pan T cell or CD8^+^ T cell MACS kit (Miltenyi Biotec) as per manufacturers’ instructions.

### MTT, Combination Index, Annexin V/7AAD apoptosis assays

For the MTT calorimetric assay, cells were seeded in 96 well plates and viability was assessed following addition of the MTT (Sigma) reagent. When assessing viability of tumor cells following co-culture experiments as done in the screen, T cells were removed prior to the MTT reagent addition. Half-maximal inhibitory concentrations (IC50) values were computed from dose–response curves using Prism (v5.0, GraphPad Software). Combination index was calculated from dose response curves of cells treated with 5-NL, Vemurafenib or in combination of two compounds at constant ratio. CompuSyn software was used to evaluate synergy using the median-effect model. For Annexin V/7AAD apoptosis assays, trypsinized cells were washed and stained in Annexin V binding buffer (BD Biosciences). Cells were analyzed for apoptosis using flow cytometry (FACS Fortessa, BD Biosciences).

### Flow cytometric analysis

Tumors or lymph nodes were excised, weighed, crushed, strained through a 0.45 micron filter and re-suspended in FACS buffer (PBS, 1% FCS, 5 mM EDTA) and surface stained with anti-CD8, CD45.2, H2DB, H2KB, KLRG1, PD1, CD95, Tim3, CD62L or CD44 (eBioscience) A full list of all antibodies is provided in Supplementary Table 4. For tetramer staining, singly suspended cells were incubated with tetramer-gp33 or tetramer-np396 (CD8) for 15 min at 37 °C. After incubation, surface antibodies (anti-CD8, anti-PD1, anti-KLRG1) were added for 30 min at 4 °C. For intracellular cytokine re-stimulation following in vivo LCMV infection, singly suspended cells were stimulated with LCMV specific peptide gp33, for 1 h after which Brefeldin A (eBiocience) was added for another 5 h incubation at 37 °C followed by staining with anti-Granzyme B, anti-IFNγ and anti-TNF-α. Staining of CD8^+^ T cells for Granzyme B and IFNγ within tumors, lymph nodes and for ex vivo co-culture systems was performed using the Foxp3 mouse Treg cell staining buffer kit (eBioscience). Experiments were performed with the BD Fortessa Cell Analyzer (BD Biosciences) or CytoFLEX (Beckman Coulter) and analyzed using FlowJo software.

#### ELISA

The IFNγ ELISA (eBioscience) was performed according to the manufacturers’ instructions.

### Immunoblotting

Cells were lysed using boiling hot SDS lysis buffer (1.1% SDS, 11% glycerol, 0.1 mol/L Tris, pH 6.8) with 10% β-mercaptoethanol. Blots were probed with anti-α-tubulin (Merck), anti-HTR1D (ThermoFischer, SantaCruz), anti-Akt, anti-p-Akt (Ser 473), anti-S6, anti-p-S6 (Ser235/236), anti-p70 S6, anti-p-p70 S6 (Thr421/Ser424), anti-p-ERK1/2, anti-ERK1/2, anti-NF-κB p65 anti-STAT-1, anti-Caspase-3, anti-Caspase-9, anti-Caspase-8, anti-AMPK and anti-p-AMPK (Thr172) (all from Cell Signaling) and detected using the Odyssey infrared imaging system (Odyssey Fc, LI-COR Biosciences). Immunoblots were quantified using ImageJ.

### Histology

Histological analysis was performed on snap frozen tissue. Tissue sections were fixed in acetone or 10% neutral buffered formalin, blocked with 5% FCS/0.3% Triton-X in PBS and stained with anti-active Caspase 3 (BD Biosciences), cleaved Caspase 8, cleaved Caspase-9, p-CREB (Ser 133), p-AMPK (Thr172) (all from Cell Signaling), CD8 and MHC-I (both from BD Biosciences) followed by incubation with the appropriate secondary antibodies. Cy3-conjugated anti-rabbit secondary antibodies were used for immunofluorescence. HRP-linked anti-rabbit secondary antibodies were used for conventional staining, which were visualised with the Peroxidase Substrate (ImmPACT NovaRED). Images were taken with an Axio Observer Z1 fluorescence microscope or Axiocam 503 color microscope (ZEISS) and quantified using Image J using the fluorescence intensity (MFI) per area as previously described [72].

### Quantitative RT-PCR

RNA was isolated using Trizol (Invitrogen). cDNA was synthetized with the Reverse Transcription System (Promega). RT-qPCR was performed using the GoTaq® qPCR mix (Promega) or the one step cDNA-qPCR (iTaq™ Universal Probes for fluorescent labeled primers or iTaq™ Universal SYBR® Green One-Step RT-qPCR Kit (Biorad)) according to the manufacturer’s instructions. A full list of primers is provided in Supplementary Table 5. For analysis, expression levels were normalized to *β-Actin* and *GAPDH* for tumor cells *or TBP2* for T cells*.*

### Mice and in vivo treatments

C57BL/6 J and NSG (the NOD.Cg-Prkdc^scid^ H2-K1^tm1Bpe^ H2-D1^tm1Bpe^ Il2rg^tm1Wjl/SzJ^) mice were maintained under specific pathogen-free conditions. 7–9 week old C57BL/6 J mice were subcutaneously injected with 5 × 10^5^ B16.GP33 or MC-38 cells. 7 days post injection, mice were randomized and treated daily for 5 consecutive days with 6.25 mg/kg 5-NL, Sumatriptan 12 mg/kg or vehicle control (2.5% DMSO in PBS). Tumors were measured using calipers and tumor volume was calculated using the following formula: (tumor length x width^2^)/2. T cell transfer experiment was performed with isolated T cells from P14 mice expressing specific anti-LCMV TCR, as previously described [29]. For infection and T cell priming 7–9 week old C57BL/6 J mice were infected intravenously with 2 × 10^5^ pfu of LCMV-Armstrong. Experiments were performed under the authorization of LANUV in accordance with German law for animal protection.

### Data mining

*CREB1, B2M, HLA A-C, IFNG and IFIT3* expression data was extracted directly from the Cancer Immunome Atlas [49].

### RNA sequencing and gene set analysis

RNA was isolated using TRIzol (Thermo Fisher Scientific) and total RNA was checked for quality on tapestation. Next, RNA was processed using the QuantSeq 3´-mRNA Library Prep/Lexogen to prepare the barcoded libraries. Sequencing of samples were performed with standard: NovaSeq 6000 mode, covering: 10 M Raw Reads (on average) with standard reads: 1 × 100 bp. Fastq files were imported into Partek Flow (Partek Incorporated). Quality analysis and quality control were performed on all reads to assess read quality and to determine the amount of trimming required (both ends: 13 bases 5′ and 1 base 3′). Trimmed reads were aligned against the mm10 genome using the STAR v2.4.1d aligner. Unaligned reads were further processed using Bowtie 2 v2.2.5 aligner. Aligned reads were combined before quantifying the expression against the ENSEMBL (release 95) database by the Partek Expectation–Maximization algorithm using the counts per million normalization. Genes with missing values and with a mean expression less than one were filtered out. Finally, statistical gene set analysis was performed using a t test to determine differential expression at the gene level (*P* < 0.05, fold change ± 1,3). Partek flow default settings were used in all analyses. Sequencing of samples was performed by NGS Core Facility—Institut for Human Genetics, Life & Brain Center, (Bonn, Germany).

### Statistical analysis

Data are expressed as mean ± S.E.M. Statistically significant differences between two groups were determined using the student’s t-test and between three or more groups, the one or two-way ANOVA was used with a post-hoc test. Values of *P* < 0.05 were considered statistically significant.

### Supplementary Information

**Additional file 1: Supplementary Figure 1.** (A,B) C57BL/6J mice were infected with 2 x 10^5^ pfu of LCMV-Armstrong. 14 days post infection, splenic LCMV-primed CD8^+^ or pan T cells were isolated and co-incubated with B16 or B16.GP33 cells for 16 hours. CD8^+^ T cells were assessed for surface KLRG1 and intracellular TNFα, Granzyme B (GZMB) and IL-2 expression by flow cytometry (*n* = 4). (B) B16.GP33 cell viability was assessed by the MTT assay following from incubation with different numbers of splenic LCMV-primed pan T cells for 24 hours (*n* = 3). The vertical dotted line indicates the concentration of T cells used in the screen (5 x 10^5^). (C) Treatment with low micromolar doses of 5-NL induced apoptosis in a time and dose-dependent manner as assessed by Annexin V/7AAD staining (*n* = 3-4). Percent apoptosis was ascertained by summing up the Annexin V+/7AAD- and Annexin V+/7AAD+ populations. (D) Representative FACS blots from (C) are shown. (E, F) C57BL/6J mice were subcutaneously injected with 5 x 10^5^ B16.GP33 cells. 7 days post-tumor injection mice were randomized into two groups and treated daily with 6.25 mg/kg of 5-NL or with vehicle for five consecutive days. Mice were sacrificed on 13 days post tumor-inoculation. Tumors were analyzed for cleaved Caspase-9, active Caspase-3 and cleaved Caspase-8 (E, top panel) using immunohistochemistry on tumor sections (representative images of tumors harvested from 4–5 mice are shown). Scale bar indicates 50 µm and slides were scored using the IHC profiler (bottom panel) (F). Cleaved Caspase-9, active Caspase-3 and pro-Caspase-8 expression from whole tumors was also analyzed using immunoblot analysis (representative images of *n* = 4 are shown, cropping is indicated by a black frame). Error bars indicate SEM; *P < 0.05 as determined by a Student´s t-test (unpaired, 2 tailed) or one-way ANOVA with a Dunnett’s post-hoc test, or a Fisher’s exact test.

**Additional file 2: Supplementary Figure 2.** 5-NL does not affect infiltration of Treg’s. (A-C) C57BL/6J mice were subcutaneously injected with 5 x 10^5^ B16.GP33 cells and 13 days post-tumor inoculation tumor infiltrates were analyzed by flow cytometry. (A) The general gating strategy for identifying CD45.2^+^ infiltrates is shown. (B) Gating strategy used for the identification of specific infiltrates from the CD45.2^+^ population is shown. (C) 7 days post-tumor injection mice were randomized into two groups and treated daily with 6.25 mg/kg of 5-NL or with vehicle for five consecutive days. Expression of GATA3 and T-Bet in CD4^+^CD25^+^FOXP3^+^ Treg cells was assessed using FACS analysis (n = 6). Error bars indicate SEM; *P < 0.05 as determined by a Student´s t-test (unpaired, 2 tailed).

**Additional file 3: Supplementary Figure 3.** 5-NL does not change the immune infiltration of transferred P14 CD45.1 CD8^+^ T cells. (A) C57BL/6J mice were treated with CD8^+^ T cell depleting antibody (anti-CD8) on days -2, -1 and 7 pre and post inoculation with 5 x 10^5^ B16.GP33 cells. T cell depletion was confirmed in the blood on day 0 and day 15 post tumor-inoculation using flow cytometry (*n* = 3-4). (B) Schematic representation of T cells transfer experiment is shown. (B,C) C57BL/6J mice expressing CD45.2 isoform were inoculated with 5 x 10^5^ B16.GP33 cells and 7 days later received 2 x 10^6^ purified splenic CD8^+^ T P14 cells expressing the CD45.1 congenic marker. 8 hours post T cell injection, mice were injected with GP33 peptide and poly(I:C) to stimulate and activate the T cells. Next day, mice were randomized into two groups and treated with 6.25 mg/kg of 5-NL or with vehicle for five consecutive days. Mice were sacrificed at day 15 post tumor inoculation and organs were FACS analyzed for number of CD45.1^+^ CD8^+^ T cells in blood, lymph nodes and tumors (*n* = 4-6). Error bars indicate SEM; *P < 0.05 as determined by a Student´s t-test (unpaired, 2 tailed).

**Additional file 4: Supplementary Figure 4.** Infection upregulates serotonin receptors (HTR) in T cells. In vivo infection with LCMV Armstrong (A) resulted in an upregulation of several serotonin receptors (HTR’s) in splenic T cells 8 days post-infection as assessed by RT-PCR (*n* = 3-4). For analysis, the expression levels of all genes were normalized to *TBP2* (∆Ct). Then gene expression values were calculated relative to naïve controls and Log2 transformed. (B) Basal levels of serotonin receptors in B16 and MC-38 cells were assessed using RT-PCR (n = 6). For analysis, the expression levels of all genes were normalized to *GAPDH* (∆Ct) and then Log2 transformed. (C) C57BL/6J mice were infected with 2 x 10^5^ pfu of LCMV Armstrong and treated with 6.25 mg/kg of 5-NL or vehicle for 5 consecutive days starting at day 1 post-infection. Cells from the blood, spleen and liver were re-stimulated with LCMV-specific gp33 epitope followed by staining for IFNγ, TNFα and GZMB in the blood 8 days post-infection and TNFα and GZMB in the blood, spleen and liver 10 days post infection using FACS analysis (n = 4-5). Tet-gp33^+^ CD8^+^ T cells in the blood were measured 8 days post-infection (n = 5). (D) C57BL/6J mice were infected with 2 x 10^5^ pfu of LCMV-Armstrong. 14 days post infection, splenic primed pan T cells were isolated and co-incubated with B16.GP33 cells pre-treated for 24 hours with 3 μM of 5-NL. KLRG1, TNFα, Granzyme B (GZMB) and IL-2 were measured on CD8^+^ T cells using flow cytometry (n = 3). Error bars indicate SEM; *P < 0.05 as determined by a Student´s t-test (unpaired, 2 tailed) or a one-way ANOVA with a Dunnett’s or a Tukey post-hoc test.

**Additional file 5:** **Supplementary Figure 5.** 5-Nonyloxytryptamine (5-NL) upregulates antigen presenting machinery and H2-Db and H2-Kb in vivo. (A, B) C57BL/6J mice were subcutaneously injected with 5 x 10^5^ B16.GP33 cells. 7 days post-tumor injection mice were randomized into two groups and treated daily with 6.25 mg/kg of 5-NL or with vehicle for five consecutive days. (A) Mice were sacrificed on day 20 post tumor-inoculation and CD45.2^-^ cells were analyzed for expression of H2-Db/Kb using flow cytometry (n = 6-8). (B) Mice were sacrificed on day 13 post tumor-inoculation and tumor infiltrates were FACS-analyzed for MHC-II expression (n = 6). (C) Treatment of B16 cells with 5-NL (3 μM) for 18 hours resulted in the upregulation of antigen presenting machinery genes *B2M* and *TAPBP* at the transcriptional level as assessed by RT-PCR. Expression was normalized to *GAPDH* (n = 4-5)*. *(D) Treatment with the indicated concentrations of another HTR4 agonist, Tegaserod for 18 hours did not induce upregulation of H2-Db/Kb in B16 cells as analyzed with flow cytometry (n = 3). (E) MHC-II (mouse cell lines) and HLA- D (human cell lines) protein expression was assessed using flow cytometry following treatment with 5-NL (5 μM for RPMI-7591 and 3 μM for MC-38, SW620, A549, MDA-MB-231, B16 and MOPC cells) for 24 hours or 18 hours for B16 cells (*n* = 5). (F, left panel) C57BL/6J mice were subcutaneously injected with 5 x 10^5^ MC-38 cells. 7 days post-tumor injection, mice were randomized into two groups and treated daily with 6.25 mg/kg of 5-NL or with vehicle for five consecutive days. Mice were sacrificed on day 13 post tumor-inoculation and tumor sections stained for MHC-I using immunofluorescence (representative images of n = 3-4 are shown, scale bar indicates 50 µm) and fluorescence signal is quantified in F, right panel. (G) B16 cells were transfected with control or HTRID targeting esiRNA. (G, left panel; immunoblot where cropped is indicated by black frame) Protein levels of HTR1D were assessed using immunoblot analysis 48 hrs post transfection. (G, right panel) 48 hours post-transfection cells were treated with 3 µM of 5-NL after which H2-Db protein levels were assessed by FACS analysis 18 hrs post 5-NL treatment (n = 6). (H) sgRNA mediated HTR1D knockout clones 1 and 2 (HTR1D KO) and control clones (CTR) in B16 cells were validated using immunoblot analysis (left panel; immunoblot cropping is indicated by a black frame). H2-Db protein expression following treatment with 3 μM of 5-NL was assessed by FACS analysis (*n* = 5-6 right panel). (I) B16 cells were transfected with control or HTR2A targeting esiRNA and *HTR2A* expression was assessed using RT-PCR 24 hours post transfection in left panel (n = 4). Expression was normalized to *GAPDH. *24 hours post esiRNA transfection, cells were treated with 3 μM of 5-NL for 18 hours and expression of H2-Db was analyzed by flow cytometry in the right panel (*n* = 3). (J) B16 cells were treated for 18 hours with 3 μM 5-NL, 10 μM Serotonin, 10 μM Asenapine or pre-treated for 30 min with Asenapine, followed by 5-NL treatment. The expression of H2-Db was measured using flow cytometry (*n* = 4). Error bars indicate SEM; *P* < 0.05 as determined by a Student´s t-test (unpaired, 2 tailed) or a 1-way ANOVA with a Tukey post-hoc test.

**Additional file 6: Supplementary Figure 6.** 5-Nonyloxytryptamine (5-NL) increases antigen presenting machinery independently of IFNγ. (A and B) C57BL/6J mice were subcutaneously injected with 5 x 10^5^ B16.GP33 cells. 7 days post-tumor injection mice were randomized into two groups and treated daily with 6.25 mg/kg of 5-NL or with vehicle for five consecutive days. Mice were sacrificed on day 13 post tumor-inoculation. (A) Intra-tumoral levels of IFNγ were determined using ELISA (n = 3-4). (B) Tumoral mRNA levels of *IFNA1, IFNB1* and *IFNG* were assessed using RT-PCR. Expression was normalized to *GAPDH *(n = 8-11). (C) B16.GP33 cells were treated with 5-NL for 18 hours and expression of *IFNA1, IFNB1* and *IFNG* was assessed using RT-PCR and expression was normalized to *GAPDH* (*n* = 5)*. *(D) Levels of STAT1 protein as assessed by immunoblot analysis are shown following treatment with 5-NL (3 μM) or murine IFNγ (5 ng/ml) at the indicated time-points and quantified in the right panel (representative immunoblot of *n* = 3 is shown; cropping is indicated by a black frame). (E) MHC-I (mouse cell lines) and HLA A-C (human cell lines) and (F) PD-L1 protein expression was assessed using flow cytometry following treatment with IFNγ or 5-NL for 24 hours (*n* = 5). Error bars indicate SEM; P < 0.05 as determined by a Student´s t-test (unpaired, 2 tailed) or a 1-way ANOVA with a Dunnett’s post-hoc test.

**Additional file 7: Supplementary Figure 7**. 5-Nonyloxytryptamine (5-NL) does not affect NF-κB signaling. (A, left panel) Levels of NF-κB p65 protein were assessed in B16 cells using immunoblot analysis following treatment with 5-NL (1.5 μM and 3 μM) at the indicated time points and quantified in right panel (representative immunoblot of n = 5-6 is shown; immunoblot cropping is indicated by a black frame). (B, left panel) Representative immunofluorescent pictures of B16 or MC-38 cells treated with 5-NL (3 µM) or TNFα (40 ng/µl) for 18 hours or 24 hours respectively and stained for phosphorylated NF-κB p65 (p-NF-κB p65, Ser-536,) are shown (representative images of n = 3-4 are shown; scale bar indicates 50 µm). Single fluorescent signal from p-NF-κB p65 was quantified in the right panel. (C, top panel) Changes in protein levels of phosphorylated ERK (p-ERK), p-Akt (Ser473), p-p70 S6 (Thr421/Ser424) and p-S6 (Ser235/6)) following treatment with 5-NL (3 and 1.5 μM) at the indicated time-points were analyzed in B16 cells using immunoblot analysis (representative immunoblots of n = 3-8 are shown; immunoblot cropping is indicated by a black frame) and quantified in C, bottom panel). Error bars indicate SEM; P < 0.05 as determined by a one or two-way ANOVA with a Dunnett’s post-hoc test.

**Additional file 8: Supplementary Figure 8** (A, left panel) Representative immunofluorescent pictures of MC-38 cells treated with 5-NL (3 µM) or forskolin (10 µM) for 24 hours and stained for phosphorylated CREB (p-CREB, Ser-133) and H2-Db are shown (representative images of n = 3-4 are shown; scale bar indicates 50 µm) and fluorescent signal is quantified in A, right panel. (B, left panel). Changes in protein levels of phosphorylated CREB (ser 133) (following treatment with 5-NL (3 and 1.5 μM) in B16 cells at the indicated time-points are shown (representative immunoblots of n = 3-7 are shown; where immunoblots are cropped is indicated by a black frame) and signal quantified in B, right panel. (C, left panel) esiRNA mediated CREB knockdown using esiRNA in B16.GP33 and MC-38 cells 48 hrs post-transfection are shown using immunoblot (n = 3; where immunoblots are cropped is indicated by a black frame). (C, right panel) Levels of apoptosis using Annexin V/7AAD staining by FACS 48 hours post esiRNA transfection are shown (n = 3-7). (D) MC-38 cells were pre-treated for 30 min with the p-CREB inhibitor 3i at the indicated doses followed by treatment with 10 µM forskolin for 24 hours. Cells were FACS-analyzed for expression of H2-Db/Kb (n = 3). (E) H2-Db/Kb protein expression as measured by FACS in MC-38 cells following 24 hours of treatment with the adenylyl cyclase activator forskolin (10 μM) is shown (n = 5). (F) Levels of apoptosis as assessed by Annexin V/7AAD staining are shown following treatment of MC-38 with 5-NL and forskolin at the indicated doses for 72 hours (n = 4-6). Percent apoptosis was ascertained by summing up the Annexin V^+^/7AAD^-^ and Annexin V^+^/7AAD^+^ populations. (G) B16 and MC38 cells were treated with the CREB inhibitor 3i (8 μM) for 18 hours. Apoptosis was assessed using Annexin V/7AAD staining (n = 5). Error bars indicate SEM; P < 0.05 as determined by a Student's t-test (unpaired, 2 tailed), one or two-way ANOVA with a Dunnett’s post-hoc test.

**Additional file 9: Supplementary Figure 9.** (A) Representative immunofluorescent pictures of B16 cells (left panel) stained for phosphorylated AMPK (p-AMPK, Thr 172, green) and DAPI (blue) detected 0.5 or 1 hour post-treatment with 3 µM of 5-NL. Scale bar indicates 50 µm. Green fluorescent signal (p-AMPK) was quantified in right panel (n = 3). Error bars indicate SEM; *P < 0.05 as determined by a one-way ANOVA with a Dunnett’s post-hoc test. (B) Transcriptomic data from melanoma samples of therapy naïve patients was mined from the Cancer Immunome Atlas. Expression of *IFIT3 *positively correlated with *HLA A-C* and *B2M*

**Additional file 10.**

**Additional file 11.**

**Additional file 12.**

**Additional file 13.**

**Additional file 14.**

## Data Availability

The dataset generated in this study are available on request from the corresponding author.

## Abbreviations

5-NL: 5-Nonyloxytryptamine
CREB: CAMP Response Element-Binding Protein
AMPK: AMP-activated protein kinase
CTL: Cytotoxic T lymphocytes
MHC-I/II: major histocompatibility complex class I/II
IFNγ: Interferon γ
TME: Tumor microenvornment
Treg: Regulatory T cells
KLRG1: Killer cell lectin-like receptor subfamily G, member 1
TIL: Tumor infiltrating leukocyte
TM: Tegaserod
PKA: Protein kinase A
AC: Adenylate cyclase
Hmgb1-ps2: High Mobility Group Box 1 pseudogene 2
DAMP: Damage-associated molecular pattern
ICD: Immunogenic cell death
GPCR: G protein-coupled receptors
APC: Antigen presenting cells
DC: Dendritic cells
HTR: Serotonin receptor
TAM: Tumor associated macrophages
LCMV: Lymphocytic choriomeningitisvirus
GZMB: Granzyme B
FasL: Fas ligand
