# Supplementary figures and images for: Unleashing T cell anti-tumor immunity: new potential for 5-Nonloxytryptamine as an agent mediating MHC-I upregulation in tumors
Source: Mol Cancer. 2023 Aug 15;22:136. doi: 10.1186/s12943-023-01833-8 (PMC10426104; doi:10.1186/s12943-023-01833-8)

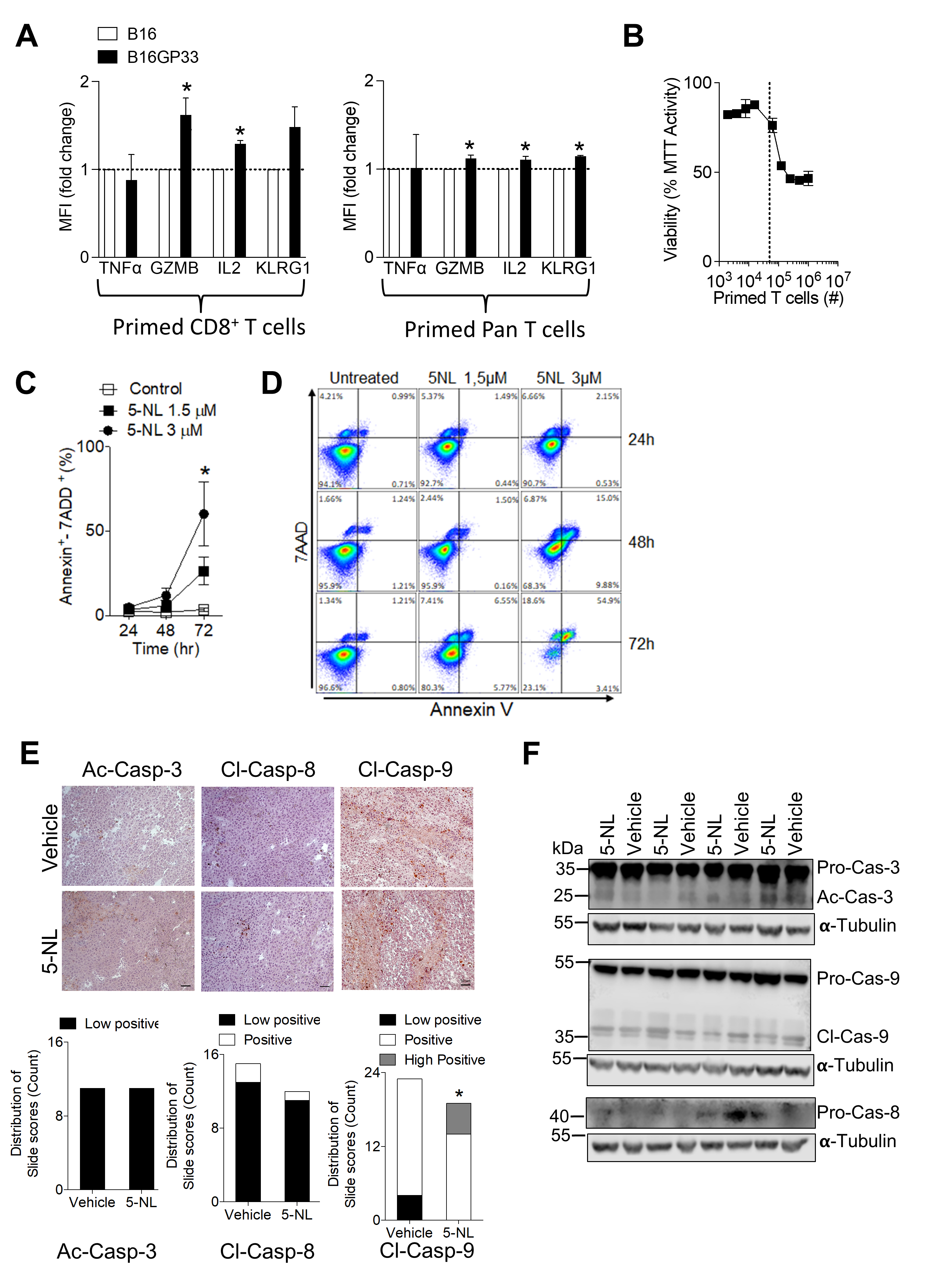

Supplement: Supplementary file 1 — Additional file 1: Supplementary Figure 1. (A,B) C57BL/6J mice were infected with 2 x 10^5^ pfu of LCMV-Armstrong. 14 days post infection, splenic LCMV-primed CD8+ or pan T cells were isolated and co-incubated with B16 or B16.GP33 cells for 16 hours. CD8+ T cells were assessed for surface KLRG1 and intracellular TNFα, Granzyme B (GZMB) and IL-2 expression by flow cytometry (n = 4). (B) B16.GP33 cell viability was assessed by the MTT assay following from incubation with different numbers of splenic LCMV-primed pan T cells for 24 hours (n = 3). The vertical dotted line indicates the concentration of T cells used in the screen (5 x 10^5^). (C) Treatment with low micromolar doses of 5-NL induced apoptosis in a time and dose-dependent manner as assessed by Annexin V/7AAD staining (n = 3-4). Percent apoptosis was ascertained by summing up the Annexin V+/7AAD- and Annexin V+/7AAD+ populations. (D) Representative FACS blots from (C) are shown. (E, F) C57BL/6J mice were subcutaneously injected with 5 x 10^5^ B16.GP33 cells. 7 days post-tumor injection mice were randomized into two groups and treated daily with 6.25 mg/kg of 5-NL or with vehicle for five consecutive days. Mice were sacrificed on 13 days post tumor-inoculation. Tumors were analyzed for cleaved Caspase-9, active Caspase-3 and cleaved Caspase-8 (E, top panel) using immunohistochemistry on tumor sections (representative images of tumors harvested from 4–5 mice are shown). Scale bar indicates 50 µm and slides were scored using the IHC profiler (bottom panel) (F). Cleaved Caspase-9, active Caspase-3 and pro-Caspase-8 expression from whole tumors was also analyzed using immunoblot analysis (representative images of n = 4 are shown, cropping is indicated by a black frame). Error bars indicate SEM; *P < 0.05 as determined by a Student´s t-test (unpaired, 2 tailed) or one-way ANOVA with a Dunnett’s post-hoc test, or a Fisher’s exact test. [file 12943_2023_1833_MOESM1_ESM.tif]

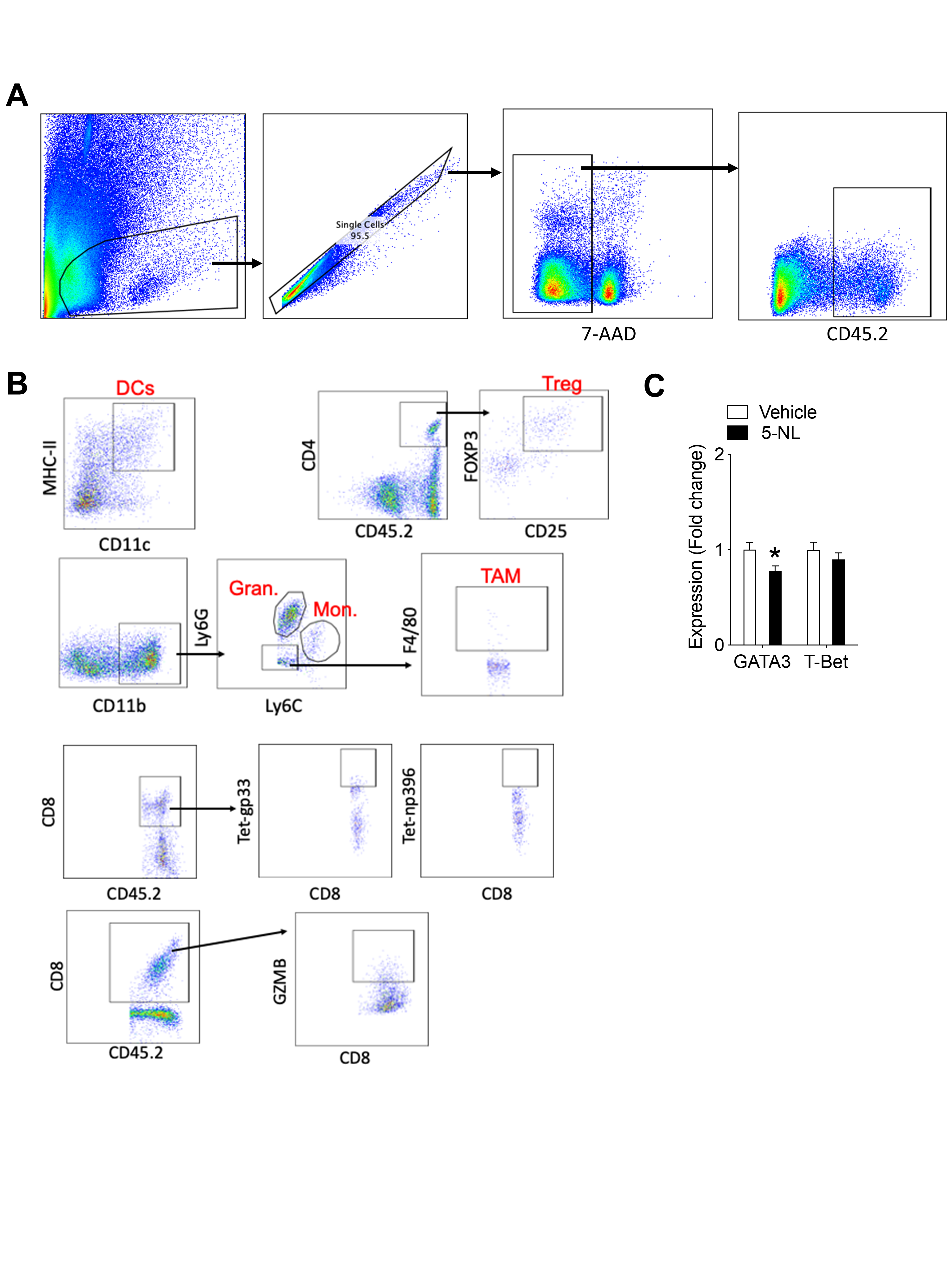

Supplement: Supplementary file 2 — Additional file 2: Supplementary Figure 2. 5-NL does not affect infiltration of Treg’s. (A-C) C57BL/6J mice were subcutaneously injected with 5 x 10^5^ B16.GP33 cells and 13 days post-tumor inoculation tumor infiltrates were analyzed by flow cytometry. (A) The general gating strategy for identifying CD45.2+ infiltrates is shown. (B) Gating strategy used for the identification of specific infiltrates from the CD45.2+ population is shown. (C) 7 days post-tumor injection mice were randomized into two groups and treated daily with 6.25 mg/kg of 5-NL or with vehicle for five consecutive days. Expression of GATA3 and T-Bet in CD4+CD25+FOXP3+ Treg cells was assessed using FACS analysis (n = 6). Error bars indicate SEM; *P < 0.05 as determined by a Student´s t-test (unpaired, 2 tailed). [file 12943_2023_1833_MOESM2_ESM.tif]

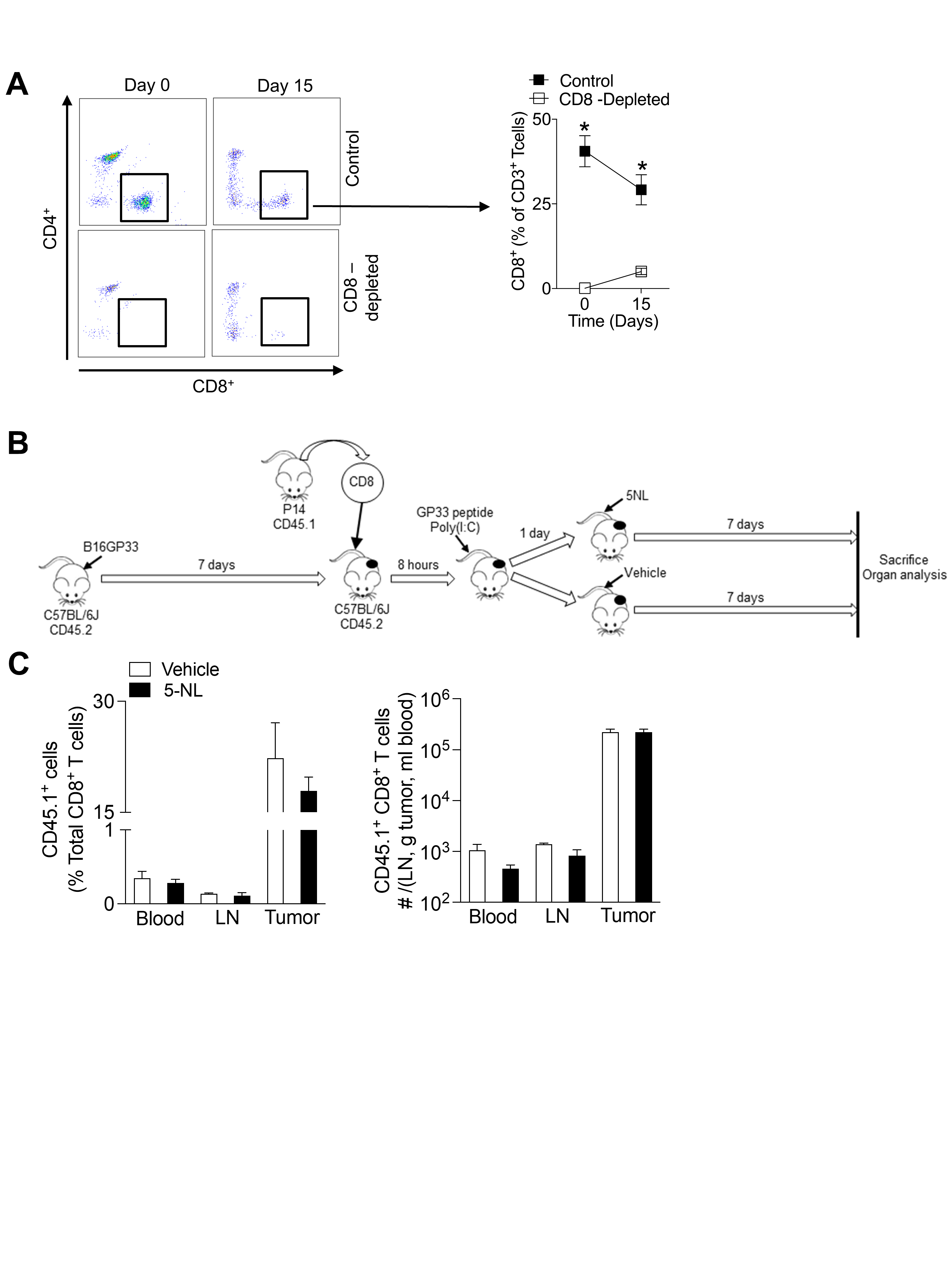

Supplement: Supplementary file 3 — Additional file 3: Supplementary Figure 3. 5-NL does not change the immune infiltration of transferred P14 CD45.1 CD8+ T cells. (A) C57BL/6J mice were treated with CD8+ T cell depleting antibody (anti-CD8) on days -2, -1 and 7 pre and post inoculation with 5 x 10^5^ B16.GP33 cells. T cell depletion was confirmed in the blood on day 0 and day 15 post tumor-inoculation using flow cytometry (n = 3-4). (B) Schematic representation of T cells transfer experiment is shown. (B,C) C57BL/6J mice expressing CD45.2 isoform were inoculated with 5 x 10^5^ B16.GP33 cells and 7 days later received 2 x 10^6^ purified splenic CD8+ T P14 cells expressing the CD45.1 congenic marker. 8 hours post T cell injection, mice were injected with GP33 peptide and poly(I:C) to stimulate and activate the T cells. Next day, mice were randomized into two groups and treated with 6.25 mg/kg of 5-NL or with vehicle for five consecutive days. Mice were sacrificed at day 15 post tumor inoculation and organs were FACS analyzed for number of CD45.1+ CD8+ T cells in blood, lymph nodes and tumors (n = 4-6). Error bars indicate SEM; *P < 0.05 as determined by a Student´s t-test (unpaired, 2 tailed). [file 12943_2023_1833_MOESM3_ESM.tif]

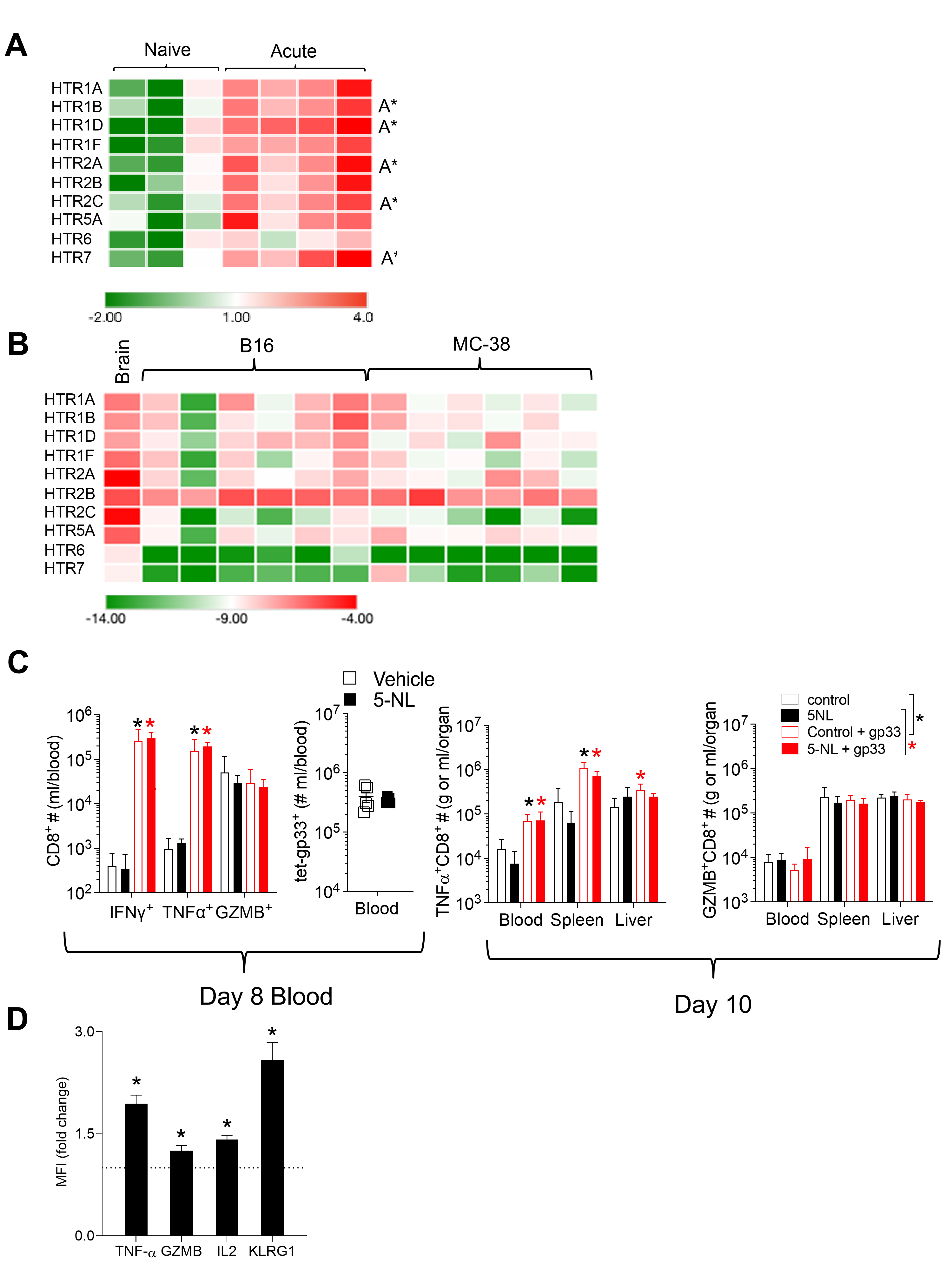

Supplement: Supplementary file 4 — Additional file 4: Supplementary Figure 4. Infection upregulates serotonin receptors (HTR) in T cells. In vivo infection with LCMV Armstrong (A) resulted in an upregulation of several serotonin receptors (HTR’s) in splenic T cells 8 days post-infection as assessed by RT-PCR (n = 3-4). For analysis, the expression levels of all genes were normalized to TBP2 (∆Ct). Then gene expression values were calculated relative to naïve controls and Log2 transformed. (B) Basal levels of serotonin receptors in B16 and MC-38 cells were assessed using RT-PCR (n = 6). For analysis, the expression levels of all genes were normalized to GAPDH (∆Ct) and then Log2 transformed. (C) C57BL/6J mice were infected with 2 x 10^5^ pfu of LCMV Armstrong and treated with 6.25 mg/kg of 5-NL or vehicle for 5 consecutive days starting at day 1 post-infection. Cells from the blood, spleen and liver were re-stimulated with LCMV-specific gp33 epitope followed by staining for IFNγ, TNFα and GZMB in the blood 8 days post-infection and TNFα and GZMB in the blood, spleen and liver 10 days post infection using FACS analysis (n = 4-5). Tet-gp33+ CD8+ T cells in the blood were measured 8 days post-infection (n = 5). (D) C57BL/6J mice were infected with 2 x 10^5^ pfu of LCMV-Armstrong. 14 days post infection, splenic primed pan T cells were isolated and co-incubated with B16.GP33 cells pre-treated for 24 hours with 3 μM of 5-NL. KLRG1, TNFα, Granzyme B (GZMB) and IL-2 were measured on CD8+ T cells using flow cytometry (n = 3). Error bars indicate SEM; *P < 0.05 as determined by a Student´s t-test (unpaired, 2 tailed) or a one-way ANOVA with a Dunnett’s or a Tukey post-hoc test. [file 12943_2023_1833_MOESM4_ESM.tif]

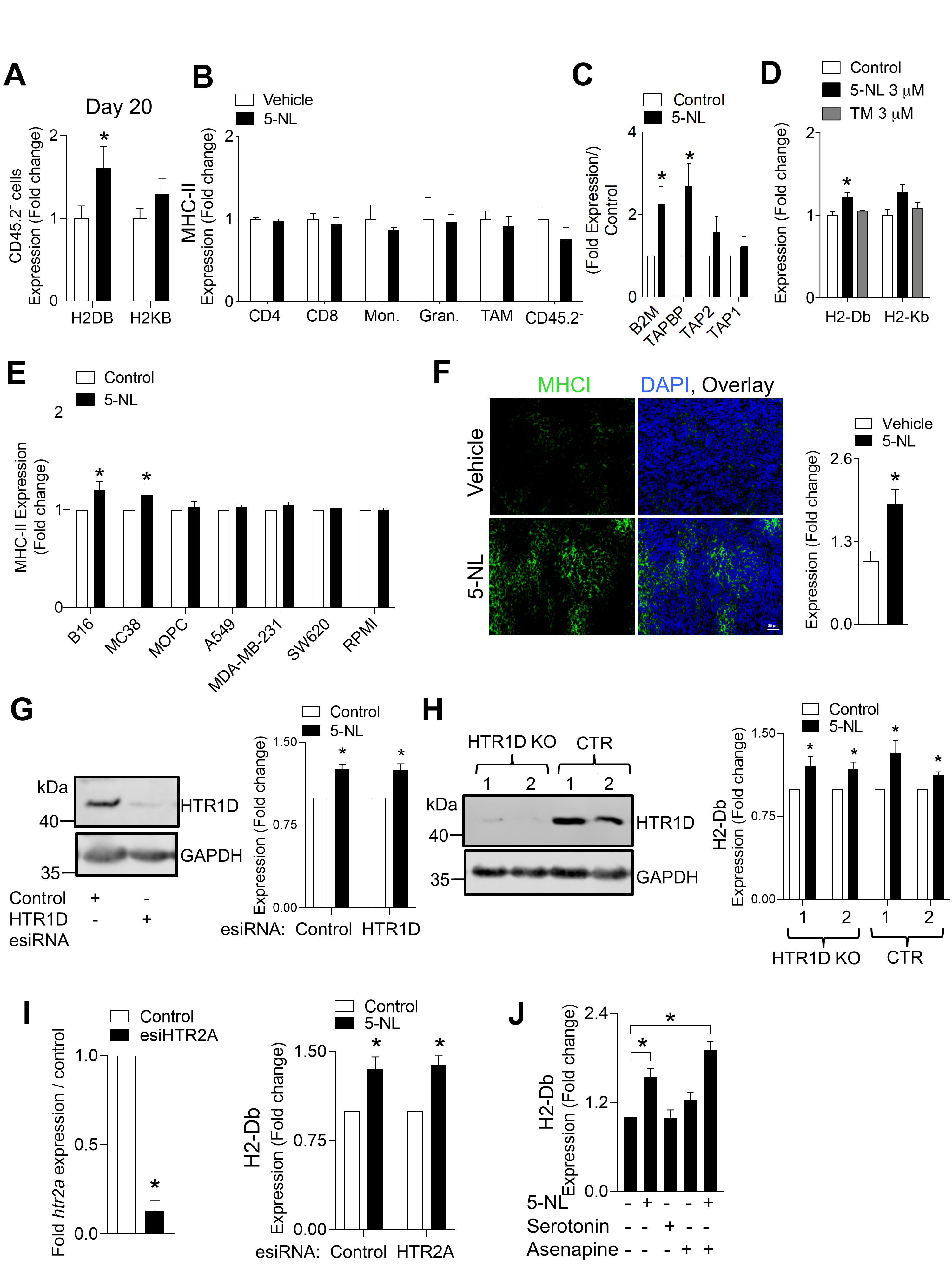

Supplement: Supplementary file 5 — Additional file 5: Supplementary Figure 5. 5-Nonyloxytryptamine (5-NL) upregulates antigen presenting machinery and H2-Db and H2-Kb in vivo. (A, B) C57BL/6J mice were subcutaneously injected with 5 x 10^5^ B16.GP33 cells. 7 days post-tumor injection mice were randomized into two groups and treated daily with 6.25 mg/kg of 5-NL or with vehicle for five consecutive days. (A) Mice were sacrificed on day 20 post tumor-inoculation and CD45.2- cells were analyzed for expression of H2-Db/Kb using flow cytometry (n = 6-8). (B) Mice were sacrificed on day 13 post tumor-inoculation and tumor infiltrates were FACS-analyzed for MHC-II expression (n = 6). (C) Treatment of B16 cells with 5-NL (3 μM) for 18 hours resulted in the upregulation of antigen presenting machinery genes B2M and TAPBP at the transcriptional level as assessed by RT-PCR. Expression was normalized to GAPDH (n = 4-5). (D) Treatment with the indicated concentrations of another HTR4 agonist, Tegaserod for 18 hours did not induce upregulation of H2-Db/Kb in B16 cells as analyzed with flow cytometry (n = 3). (E) MHC-II (mouse cell lines) and HLA- D (human cell lines) protein expression was assessed using flow cytometry following treatment with 5-NL (5 μM for RPMI-7591 and 3 μM for MC-38, SW620, A549, MDA-MB-231, B16 and MOPC cells) for 24 hours or 18 hours for B16 cells (n = 5). (F, left panel) C57BL/6J mice were subcutaneously injected with 5 x 10^5^ MC-38 cells. 7 days post-tumor injection, mice were randomized into two groups and treated daily with 6.25 mg/kg of 5-NL or with vehicle for five consecutive days. Mice were sacrificed on day 13 post tumor-inoculation and tumor sections stained for MHC-I using immunofluorescence (representative images of n = 3-4 are shown, scale bar indicates 50 µm) and fluorescence signal is quantified in F, right panel. (G) B16 cells were transfected with control or HTRID targeting esiRNA. (G, left panel; immunoblot where cropped is indicated by black frame) Protein levels of HTR1D wer [file 12943_2023_1833_MOESM5_ESM.tif]

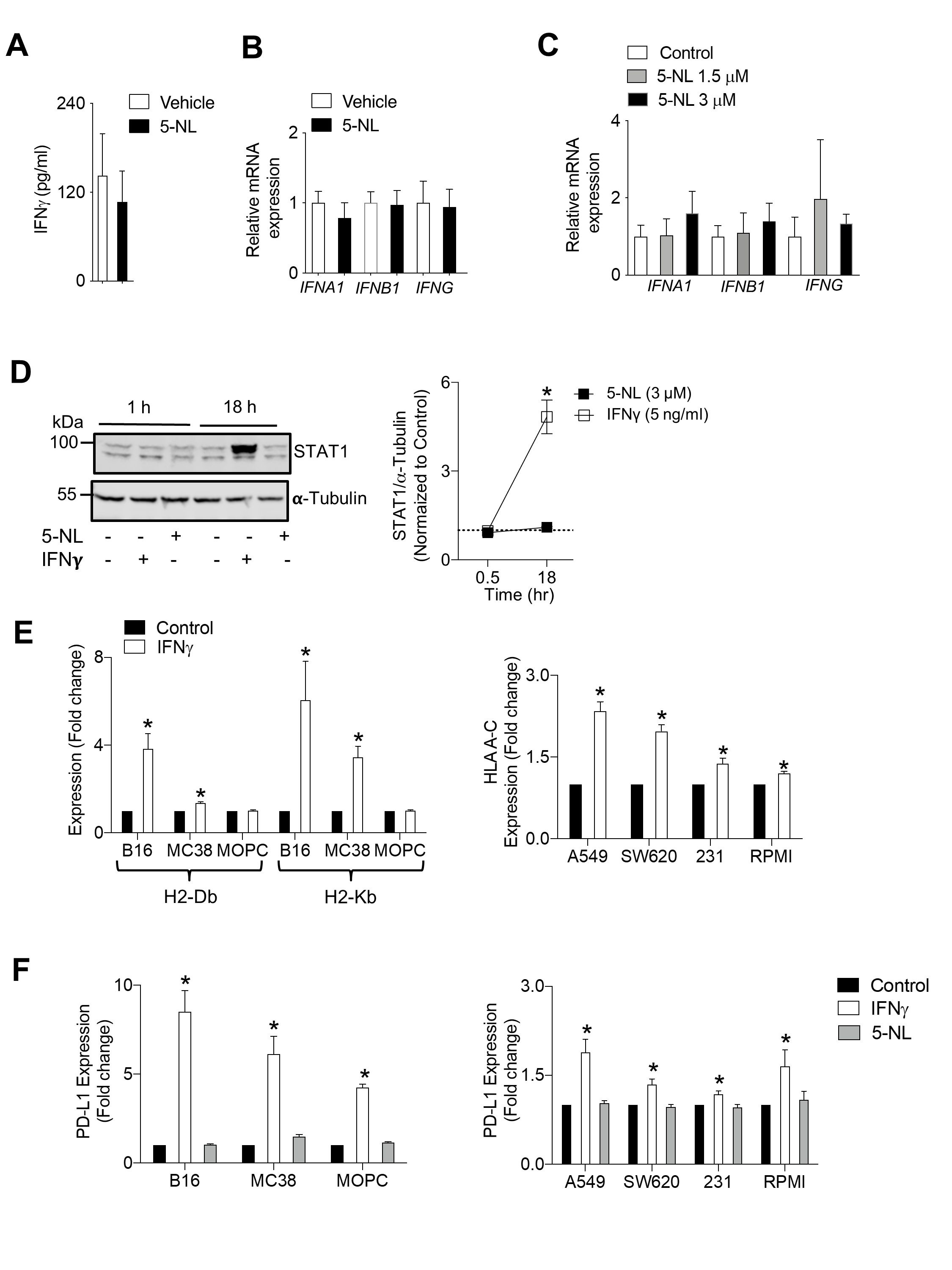

Supplement: Supplementary file 6 — Additional file 6: Supplementary Figure 6. 5-Nonyloxytryptamine (5-NL) increases antigen presenting machinery independently of IFNγ. (A and B) C57BL/6J mice were subcutaneously injected with 5 x 10^5^ B16.GP33 cells. 7 days post-tumor injection mice were randomized into two groups and treated daily with 6.25 mg/kg of 5-NL or with vehicle for five consecutive days. Mice were sacrificed on day 13 post tumor-inoculation. (A) Intra-tumoral levels of IFNγ were determined using ELISA (n = 3-4). (B) Tumoral mRNA levels of IFNA1, IFNB1 and IFNG were assessed using RT-PCR. Expression was normalized to GAPDH (n = 8-11). (C) B16.GP33 cells were treated with 5-NL for 18 hours and expression of IFNA1, IFNB1 and IFNG was assessed using RT-PCR and expression was normalized to GAPDH (n = 5). (D) Levels of STAT1 protein as assessed by immunoblot analysis are shown following treatment with 5-NL (3 μM) or murine IFNγ (5 ng/ml) at the indicated time-points and quantified in the right panel (representative immunoblot of n = 3 is shown; cropping is indicated by a black frame). (E) MHC-I (mouse cell lines) and HLA A-C (human cell lines) and (F) PD-L1 protein expression was assessed using flow cytometry following treatment with IFNγ or 5-NL for 24 hours (n = 5). Error bars indicate SEM; P < 0.05 as determined by a Student´s t-test (unpaired, 2 tailed) or a 1-way ANOVA with a Dunnett’s post-hoc test. [file 12943_2023_1833_MOESM6_ESM.tif]

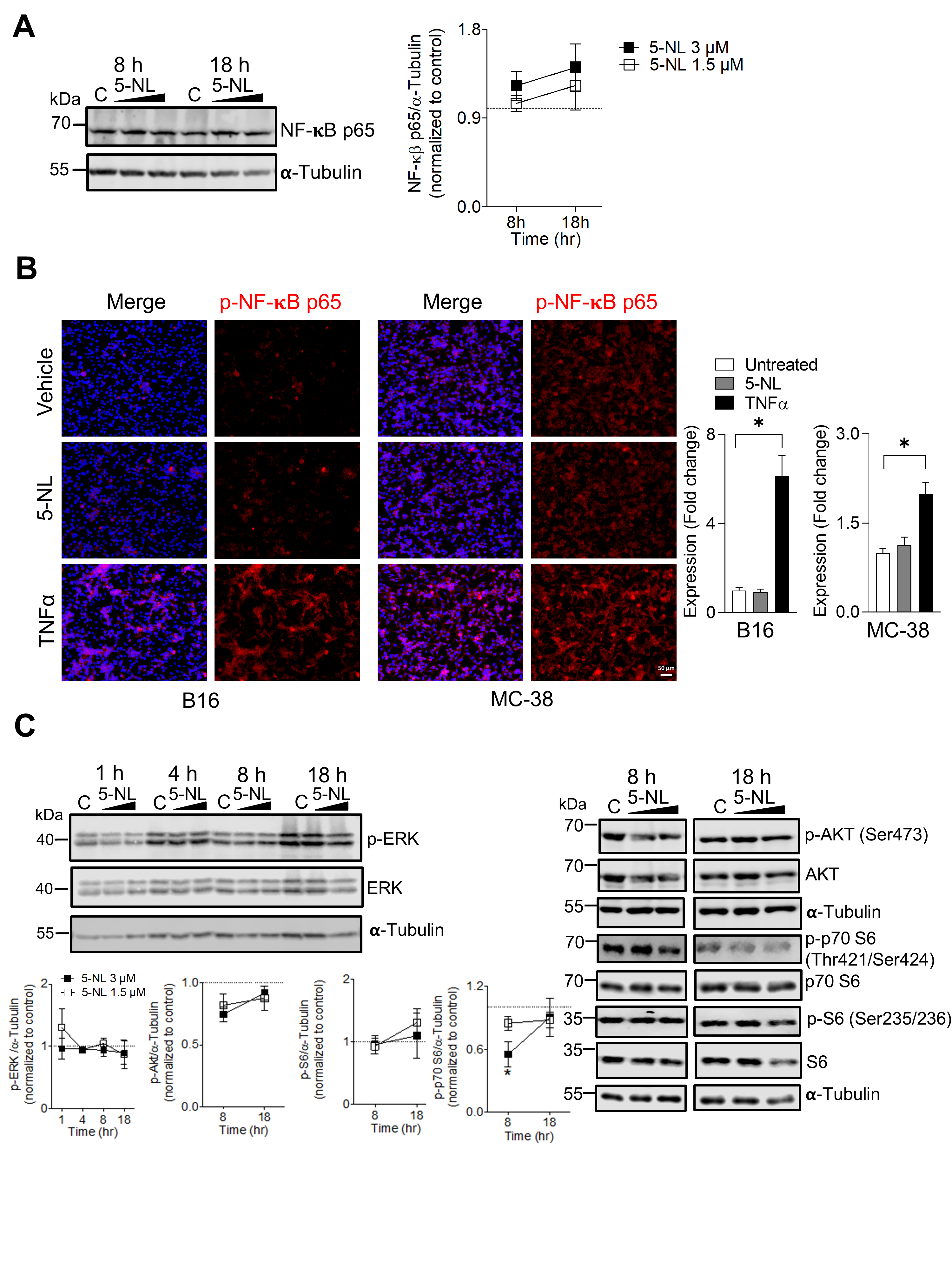

Supplement: Supplementary file 7 — Additional file 7: Supplementary Figure 7. 5-Nonyloxytryptamine (5-NL) does not affect NF-κB signaling. (A, left panel) Levels of NF-κB p65 protein were assessed in B16 cells using immunoblot analysis following treatment with 5-NL (1.5 μM and 3 μM) at the indicated time points and quantified in right panel (representative immunoblot of n = 5-6 is shown; immunoblot cropping is indicated by a black frame). (B, left panel) Representative immunofluorescent pictures of B16 or MC-38 cells treated with 5-NL (3 µM) or TNFα (40 ng/µl) for 18 hours or 24 hours respectively and stained for phosphorylated NF-κB p65 (p-NF-κB p65, Ser-536,) are shown (representative images of n = 3-4 are shown; scale bar indicates 50 µm). Single fluorescent signal from p-NF-κB p65 was quantified in the right panel. (C, top panel) Changes in protein levels of phosphorylated ERK (p-ERK), p-Akt (Ser473), p-p70 S6 (Thr421/Ser424) and p-S6 (Ser235/6)) following treatment with 5-NL (3 and 1.5 μM) at the indicated time-points were analyzed in B16 cells using immunoblot analysis (representative immunoblots of n = 3-8 are shown; immunoblot cropping is indicated by a black frame) and quantified in C, bottom panel). Error bars indicate SEM; P < 0.05 as determined by a one or two-way ANOVA with a Dunnett’s post-hoc test. [file 12943_2023_1833_MOESM7_ESM.tif]

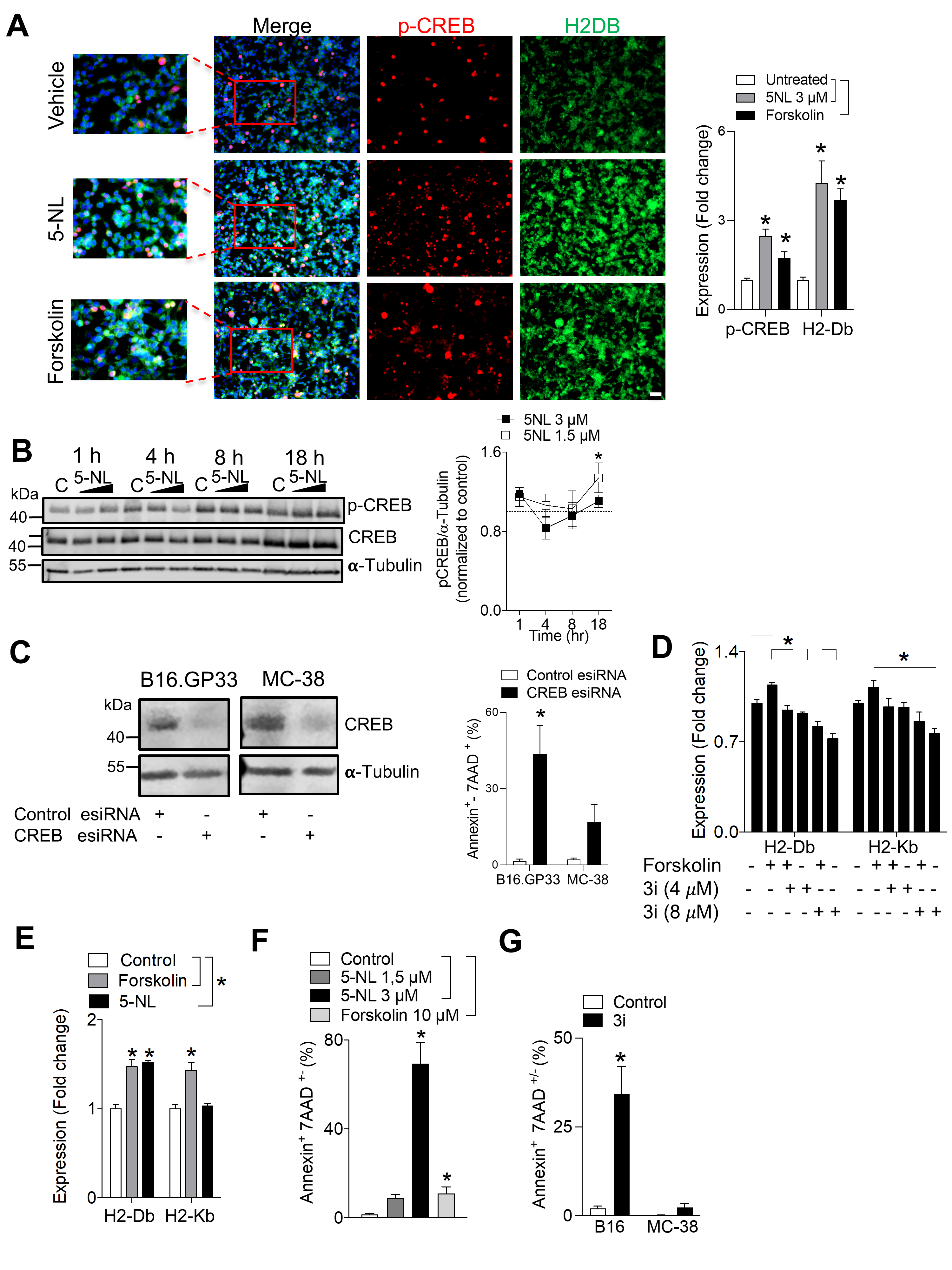

Supplement: Supplementary file 8 — Additional file 8: Supplementary Figure 8 (A, left panel) Representative immunofluorescent pictures of MC-38 cells treated with 5-NL (3 µM) or forskolin (10 µM) for 24 hours and stained for phosphorylated CREB (p-CREB, Ser-133) and H2-Db are shown (representative images of n = 3-4 are shown; scale bar indicates 50 µm) and fluorescent signal is quantified in A, right panel. (B, left panel). Changes in protein levels of phosphorylated CREB (ser 133) (following treatment with 5-NL (3 and 1.5 μM) in B16 cells at the indicated time-points are shown (representative immunoblots of n = 3-7 are shown; where immunoblots are cropped is indicated by a black frame) and signal quantified in B, right panel. (C, left panel) esiRNA mediated CREB knockdown using esiRNA in B16.GP33 and MC-38 cells 48 hrs post-transfection are shown using immunoblot (n = 3; where immunoblots are cropped is indicated by a black frame). (C, right panel) Levels of apoptosis using Annexin V/7AAD staining by FACS 48 hours post esiRNA transfection are shown (n = 3-7). (D) MC-38 cells were pre-treated for 30 min with the p-CREB inhibitor 3i at the indicated doses followed by treatment with 10 µM forskolin for 24 hours. Cells were FACS-analyzed for expression of H2-Db/Kb (n = 3). (E) H2-Db/Kb protein expression as measured by FACS in MC-38 cells following 24 hours of treatment with the adenylyl cyclase activator forskolin (10 μM) is shown (n = 5). (F) Levels of apoptosis as assessed by Annexin V/7AAD staining are shown following treatment of MC-38 with 5-NL and forskolin at the indicated doses for 72 hours (n = 4-6). Percent apoptosis was ascertained by summing up the Annexin V+/7AAD- and Annexin V+/7AAD+ populations. (G) B16 and MC38 cells were treated with the CREB inhibitor 3i (8 μM) for 18 hours. Apoptosis was assessed using Annexin V/7AAD staining (n = 5). Error bars indicate SEM; P < 0.05 as determined by a Student's t-test (unpaired, 2 tailed), one or two-way ANOVA with a Dunnett’s post-hoc test. [file 12943_2023_1833_MOESM8_ESM.tif]

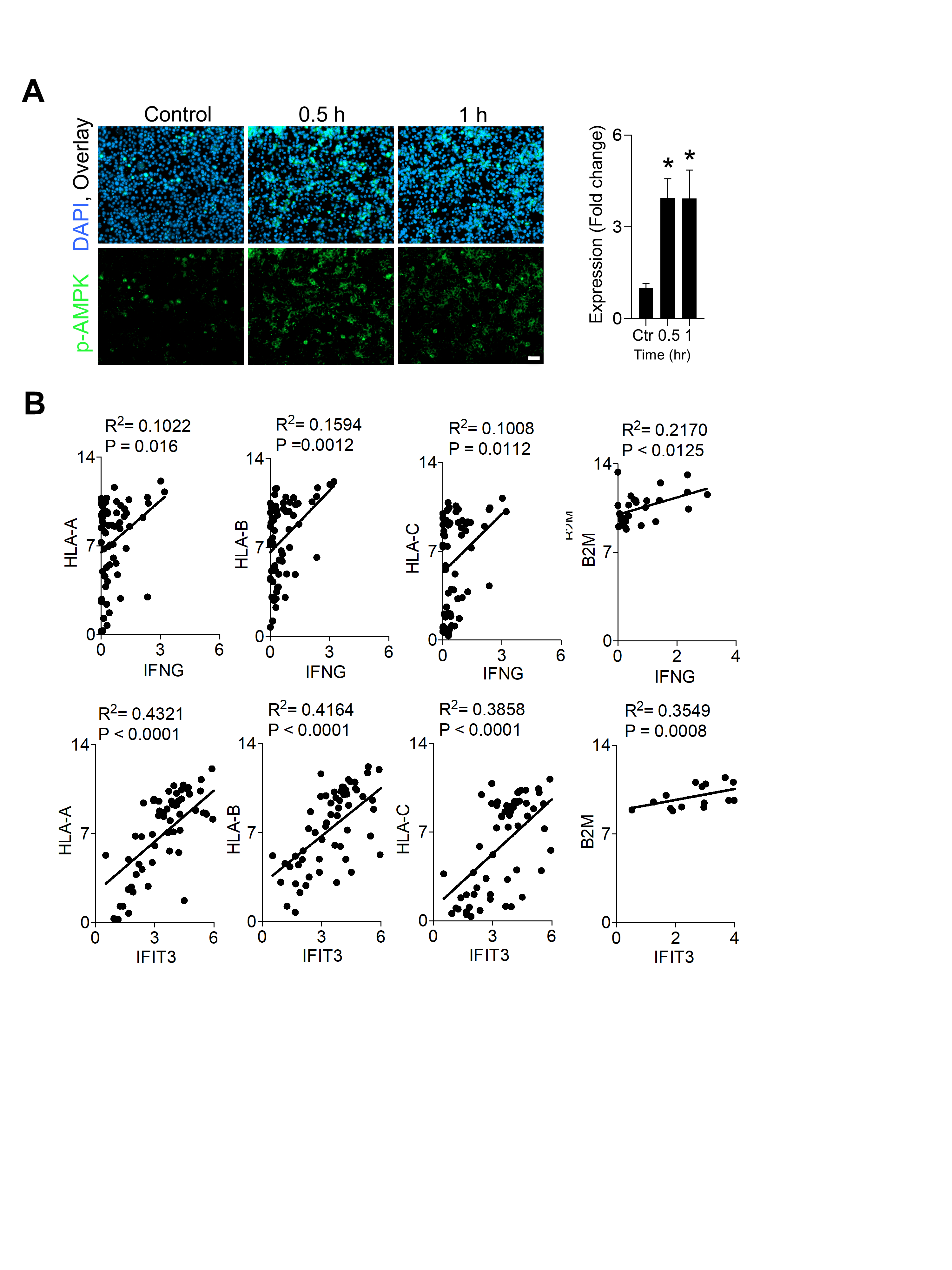

Supplement: Supplementary file 9 — Additional file 9: Supplementary Figure 9. (A) Representative immunofluorescent pictures of B16 cells (left panel) stained for phosphorylated AMPK (p-AMPK, Thr 172, green) and DAPI (blue) detected 0.5 or 1 hour post-treatment with 3 µM of 5-NL. Scale bar indicates 50 µm. Green fluorescent signal (p-AMPK) was quantified in right panel (n = 3). Error bars indicate SEM; *P < 0.05 as determined by a one-way ANOVA with a Dunnett’s post-hoc test. (B) Transcriptomic data from melanoma samples of therapy naïve patients was mined from the Cancer Immunome Atlas. Expression of IFIT3 positively correlated with HLA A-C and B2M [file 12943_2023_1833_MOESM9_ESM.tif]
